# Functional abnormalities in induced Pluripotent Stem Cell-derived cardiomyocytes generated from titin-mutated patients with dilated cardiomyopathy

**DOI:** 10.1371/journal.pone.0205719

**Published:** 2018-10-17

**Authors:** Revital Schick, Lucy N. Mekies, Yuval Shemer, Binyamin Eisen, Tova Hallas, Ronen Ben Jehuda, Meital Ben-Ari, Agnes Szantai, Lubna Willi, Rita Shulman, Michael Gramlich, Luna Simona Pane, Ilaria My, Dov Freimark, Marta Murgia, Gianluca Santamaria, Mihaela Gherghiceanu, Michael Arad, Alessandra Moretti, Ofer Binah

**Affiliations:** 1 Department of Physiology, Biophysics and Systems Biology, Technion, Haifa, Israel; 2 The Rappaport Institute, Haifa, Israel; 3 Rappaport Faculty of Medicine, Technion, Haifa, Israel; 4 Department of Biotechnology, Technion, Haifa, Israel; 5 Department of Biochemistry, University of Szeged, Szeged, Hungary; 6 Department of Cardiology and Cardiovascular Diseases, Eberhard Karls University, Tübingen, Germany; 7 Discovery Biology, Discovery Sciences, IMED Biotech Unit, AstraZeneca, Gothenburg, Sweden; 8 Medical Department–Cardiology, Klinikum rechts der Isar–Technische Universität München, Munich, Germany; 9 Heart Failure Institute and Leviev Heart Center, Sheba Medical Center and Sackler School of Medicine, Tel Aviv University, Tel Aviv, Israel; 10 Department of Proteomics and Signal Transduction, Max-Planck-Institute of Biochemistry, Martinsried, Germany; 11 Department of Biomedical Sciences, University of Padova, Padua, Italy; 12 Department of Experimental and Clinical Medicine, University of Magna Grecia, Medical School, Catanzaro, Italy; 13 Victor Babes National Institute of Pathology, Bucharest, Romania; 14 German Centre for Cardiovascular Research–partner site Munich Heart Alliance, Munich, Germany; University of Tampere, FINLAND

## Abstract

**Aims:**

Dilated cardiomyopathy (DCM), a myocardial disorder that can result in progressive heart failure and arrhythmias, is defined by ventricular chamber enlargement and dilatation, and systolic dysfunction. Despite extensive research, the pathological mechanisms of DCM are unclear mainly due to numerous mutations in different gene families resulting in the same outcome—decreased ventricular function. Titin (*TTN*)—a giant protein, expressed in cardiac and skeletal muscles, is an important part of the sarcomere, and thus *TTN* mutations are the most common cause of adult DCM. To decipher the basis for the cardiac pathology in titin-mutated patients, we investigated the hypothesis that induced Pluripotent Stem Cell (iPSC)-derived cardiomyocytes (iPSC-CM) generated from patients, recapitulate the disease phenotype. The hypothesis was tested by 3 Aims: (1) Investigate key features of the excitation-contraction-coupling machinery; (2) Investigate the responsiveness to positive inotropic interventions; (3) Investigate the proteome profile of the AuP cardiomyocytes using mass-spectrometry (MS).

**Methods and results:**

iPSC were generated from the patients' skin fibroblasts. The major findings were: (1) Sarcomeric organization analysis in mutated iPSC-CM showed defects in assembly and maintenance of sarcomeric structure. (2) Mutated iPSC-CM exhibited diminished inotropic and lusitropic responses to β-adrenergic stimulation with isoproterenol, increased [Ca^2+^]_out_ and angiotensin-II. Additionally, mutated iPSC-CM displayed prolonged recovery in response to caffeine. These findings may result from defective or lack of interactions of the sarcomeric components with titin through its kinase domain which is absent in the mutated cells.

**Conclusions:**

These findings show that the mutated cardiomyocytes from DCM patients recapitulate abnormalities of the inherited cardiomyopathies, expressed as blunted inotropic response.

## Introduction

Dilated cardiomyopathy (DCM), the most common cardiomyopathy, is a myocardial disorder defined by ventricular chamber enlargement and dilatation, and systolic dysfunction that can result in progressive heart failure, supraventricular and ventricular arrhythmias. Consequently, DCM is a major cause of morbidity and mortality contributing significantly to health care costs [1–3]. The prevalence and incidence of idiopathic DCM were estimated to be 1:2500 individuals and 6–7:100,000 respectively, but the data collected was based on outdated diagnosis methods. In 20–50% of cases the disease is inherited and is referred as familial DCM [2,3]. The most common inheritance form of DCM is autosomal dominant transmission, although other forms were described, such as autosomal recessive, X-linked and mitochondrial inheritance [4].

Despite extensive research in recent years, the complex pathological mechanisms of DCM are still unclear, mainly because numerous mutations in different gene families result in a similar outcome—depressed ventricular function. Hence, mutations in nuclear, sarcomeric, cytoskeletal and surface membrane, impair myocardial force generation and relaxation, force transmission, and/or cell survival. Furthermore, even when a mutation is identified, the link between the genetic abnormality and cardiac dysfunction is often unclear [5].

Titin, the largest sarcomeric protein, is expressed in cardiac and skeletal muscles and is encoded by the *TTN* gene. The titin protein is located within the sarcomere as a third filament around 1 μm in length connecting between Z-line and M-line [2,3]. Titin functions as a molecular bi-directional spring responsible for the passive elasticity of the muscle, by creating a restoring force that causes the sarcomere to return to its resting length [6,7]. Mutations in titin are a frequent cause of DCM [8], and were described in several families inherited as an autosomal dominant trait.

To decipher the cellular basis for the cardiac pathology in titin-mutated patients, we investigated the hypothesis that iPSC-CM generated from titin-mutated DCM patients recapitulate key aspects of the disease phenotype. To this end, we generated iPSC-CM from two DCM patients [9,10] carrying different mutations in the *TTN* gene, and investigated their excitation-contraction coupling (ECC) machinery and responsiveness to common positive inotropic interventions. The present study demonstrates that while the basal ECC machinery of titin-mutated iPSC-CM appears intact, their responsiveness to positive inotropic interventions is blunted compared to healthy iPSC-CM.

## Methods

http://dx.doi.org/10.17504/protocols.io.tvien4e

### Generation of patient-specific induced pluripotent stem cells

Skin biopsies were acquired according to approval #3611 issued by the Helsinki Committee for experiments on human subjects at the Rambam Health Care Campus, Haifa, Israel. The biopsy [human dermal fibroblasts (HDF)] was obtained from a 28-year-old DCM patient (denoted IsP) from an Israeli family carrying an adenine insertion mutation, causing a frame shift and resulting in a stop codon and protein truncation after 19,628 amino acids [9]. Additional skin (HDF24) and hair (KTN3 and KTI [11,12]) biopsies were obtained from healthy subjects as control. The dermal fibroblasts generated from the skin biopsies were reprogrammed as previously described [13] using the STEMCCA Cassette (a single lentiviral vector containing the four factors: Oct4, Sox2, Klf4 and c-Myc) and induced Pluripotent Stem Cells (iPSC) were generated (IsP DCM clones 23.2 and 23.10). These iPSC clones were spontaneously differentiated into functional cardiomyocytes as previously described [11–13].

### Karyotype analysis

Karyotype analysis was performed using standard G-banding chromosome analysis by the cytogenetic laboratory according to standard procedures.

### Teratoma formation

To evaluate the iPSC differentiation capacity *in vivo*, 5-day-old iPSC colonies from one 6-well plate were detached using 1 mg/ml type IV collagenase, washed 3 times in PBS and then injected into thigh muscle of severe combined immunodeficient (SCID) mice. Teratomas were observed 8–12 weeks after injection, and images were obtained from formalin-fixed (4%) and paraffin-embedded teratoma sections stained with hematoxylin and eosin (H&E) [14–16].

### Genotyping

To confirm that the mutation is preserved in the iPSC clones, sequence analysis was performed on the titin gene both in the patients-derived iPSC and fibroblasts by performing PCR using primers which delimit the mutation area in the mutated gene. The PCR was performed on genomic DNA produced from the patients' fibroblasts and iPSC using Promega DNA purification kit, with the primers: F-5’-TATTGCCTGGGTTAAGCCGC-3’ and R-5’-AGCTCCTGTTGTTAGTCCGC-3’.

### Immunofluorescence staining

Immunofluorescence staining was performed according to standard protocols using the following antibodies: Alexa Fluor 555 donkey anti-rabbit (1:100; Life Technologies, Eugene, OR, USA), Alexa Fluor 488 donkey anti mouse (1:100; Life Technologies, Eugene, OR, USA), cy5 donkey anti goat (1:100; Invitrogene, Eugene, Oregon, USA), OCT3/4 (1:100; Millipore, Santa Cruz, CA, USA), SSEA4 (1:100; Millipore, Temecula, CA, USA), TRA1-60 (1:100; Millipore, Temecula, CA, California), Nanog (1:50; R&D, Minneapolis, MN, USA), DAPI (1:500; Sigma Aldrich, St. Louis, MO, USA).

### Action potential recordings

For action potentials (AP) recordings, spontaneously contracting areas of EBs (control data was obtained from experiments on KTI and KTN3 clones used in Ben-Ari et al [11]) were mechanically and/or enzymatically dispersed (collagenase II 1 mg/ml; Worthington, Lakewood, New Jersey, USA, http://www.worthington-biochem.com). Small clusters were then plated on gelatin-coated glass coverslips (13 mm diameter) in 24-well plates. The coverslips were incubated at 37°C, and a recovery period of at least two days was allowed before the electrophysiological experiment was performed [11]. In all experiments, the coverslips were perfused at 37°C with an external solution containing (in mM): 140 NaCl, 5.4 KCl, 1.8 CaCl_2_, 1 MgCl_2_, 10 glucose and 10 HEPES titrated to pH 7.4 with NaOH. The patch pipette solution contained (mM): 120 KCl, 1 MgCl_2_, 3 Mg-ATP, 10 HEPES, and 10 EGTA titrated to pH 7.2 with KOH and adjusted at 290 mOsm with saccharose (all materials were purchased from Sigma-Aldrich). Axopatch 200B, Digidata 1322 and pClamp10 (Molecular Devices, Sunnyvale, CA) were used for data amplification, acquisition and analysis. Signals were digitized at 10 kHz and filtered at 2 kHz. Microelectrodes with resistances of 4–7 MΩ were pulled from borosilicate glass capillaries (Harvard Apparatus, Holliston, USA). Analysis was preformed using MATLAB software (MathWorks, Natick, MA, USA). Corrected AP duration (APD) was calculated by the Bazett's correction ($cAPD=\frac{APD}{\sqrt{\frac{60}{beat\;rate}}}$).

### Extracellular electrograms recorded from spontaneously contracting EBs and analysis of Beat Rate Variability (BRV)

Extracellular electrograms were recorded from spontaneously contracting 30–60 day-old EBs using the Micro-Electrode-Array (MEA) apparatus (Multi Channels Systems, Reutlingen, Germany) routinely used in our lab [11,17]. The MEA set-up consists of a 50×50-mm glass substrate, in the center of which is embedded a 1.4×1.4 mm matrix of 60 titanium-nitride electrodes. The electrode diameter is 30 µm and inter-electrode distance is 200 µm. Spontaneously contracting EBs were mechanically dissected and adhered onto the MEA dish and their electrical activity was recorded by the MEA data acquisition software at sampling rate of 1000 Hz which was down-sampled to 200 Hz. During the recording, the cultures were kept in a bath-like conformation of a glass cylinder (glued to the center of the MEA plane) filled with 500 µL of culture medium saturated with a gas mixture consisting of 5% CO_2_ + 95% air. The temperature was kept at 37°C using a heating element and a temperature controller.

For BRV analysis, all recordings were analyzed to detect peaks of the activation spikes, from which inter-beat intervals (denoted ‘IBI’) were calculated using MATLAB software (MathWorks, Natick, MA, USA). To generate Poincaré plots, each R-R interval (IBI_n+1_) is plotted against its predecessor (IBI_n_), creating a scattered mass of points in a two-dimensional array. Quantitative analysis of the plot is performed by fitting an ellipse to the group of points, with its center coinciding with the centroid of the ellipse (the point of the average IBI), and adjusting two perpendicular lines traversing the centroid. The longitudinal line designated SD2, represents long-term variability of the data (reflecting the standard deviation of the IBIs. The perpendicular line designated SD1 represents short-term beat-to-beat variability [18].

### Measurements of intracellular Ca^2+^ transients and contractions

Intracellular Ca^2+^ ([Ca^2+^]_i_) transients and contractions were recorded from small contracting 40-70-day old embryoid bodies (EBs) by means of fura-2 fluorescence and video edge detector, respectively, using the IonOptix Calcium and Contractility system (Westwood, MA, USA) as previously described [19–21]. In brief, spontaneously contracting EBs were mechanically dissected and adhered onto 18 mm diameter gelatin-coated glass slides. Subsequently, fura-2-stained (2.5 μM) contracting areas were transferred to a chamber mounted on the stage of an inverted microscope and perfused at a rate of 1–1.5 ml/min Tyrode’s solution at 37°C. The Tyrode’s solution contains (mmol/l): 140 NaCl, 5.4 KCl, 1 MgCl_2_, 2 sodium pyrovate, 1 CaCl_2_, 10 HEPES, 10 glucose (pH 7.4 adjusted with NaOH). The EBs were paced at 0.5–2.5 Hz which corresponded to a frequency 20–50% higher than the spontaneous beating rate. The acquisition rate of both the [Ca^2+^]_i_ transients and contractions was 100 points/sec. Analysis was performed using the IonOptix designated system. To characterize the [Ca^2+^]_i_ transients amplitude, the differences between maximal (systolic) and minimal (diastolic) ratio were calculated in 20 successive transients and averaged (R_Amp_). Similarly, the contraction amplitude (L_Amp_) was calculated from the average differences between minimal and maximal video cursor positions of 20 successive contractions. In addition, the maximal rates of [Ca^2+^]_i_ rise (+d[Ca^2+^]_i_/dt) and decay (-d[Ca^2+^]_i_/dt_Relax_), and maximal rates of contraction (dL/dt_Contraction_) and relaxation (dL/dt_Relaxation_) were calculated and averaged over 20 [Ca^2+^]_i_ transients and contractions, respectively. The data from the Israeli iPSC-derived cardiomyocytes (iPSC-CM) are pulled from two clones.

### Immunocytological analysis in iPSC-CM

iPSC-CM were fixed in 3.7% (vol/vol) formaldehyde and subjected to immunostaining as previously reported [13]. Sarcomere structure was visualized using primary antibodies against cardiac troponin T (mouse monoclonal clone 13–11, Lab Vision, 1:500) and α-actinin (mouse monoclonal clone EA-53, Sigma-Aldrich, 1:300). Alexa-Fluor-488, and -594 conjugated secondary antibodies specific to the appropriate species were used (Life Technologies, 1:500). Nuclei were detected with 1 µg/ml Hoechst 33528. Microscopy was performed using imaging systems (DMI6000-AF6000), filter cubes and software from Leica microsystems. Images were assigned with pseudo-colors. Morphological analyses were performed by investigators blinded to the genotype of the cells.

### Mass Spectrometry sample preparation and data processing

iPSC-CM were resuspended in a lysis buffer containing 6M guanidinium chloride (GCl), boiled for 5 minutes and subsequently reduced with 10 mM tris (2-carboxyethyl) phosphine (TCEP) and alkylated with 40 mM 2-chloro-N,N-diethylacetamide. Samples were diluted (10% acetonitrile, 25 mM Tris pH 8.5), first 1:3 Lysate: Buffer for LysC digestion (25°C 3 hours), then 1:10 for Trypsin digestion. Samples were incubated at 37°C overnight under continuous shaking. The digestion was blocked by acidifying the sample with TFA (1% total). Peptides were de-salted using SDB-RPS StageTips as described [22]. Mass-spectrometry (MS) analysis was performed in triplicates using a nanoflow uHPLC system (Easy1000 nLC,) coupled via a nanoelectrospray ion source to a Q Exactive mass spectrometer (all from Thermo Fisher Scientific). Peptides were separated on a 50 cm long column with 75 μm inner diameter, packed in-house with ReproSil-Pur C18-AQ 1.9 μm resin (Dr. Maisch GmbH). Column temperature was kept at 50°C. Peptide separation was carried out by loading the peptides in buffer A (0.1% (v/v) formic acid) and eluting them in 120 or 240 minutes with a nonlinear gradient of 5–60% buffer B (0.1% (v/v) formic acid, 80% (v/v) acetonitrile) at a flow rate of 250 nl/min. MS analysis of peptides was performed in a data-dependent acquisition mode, with survey scans (300–1700 m/z, maximum ion injection times 60 ms) acquired at a resolution of 60,000 followed by higher-energy collisional dissociation (HCD) based fragmentation of up to 15 most abundant precursor ions. The MS/MS scans were acquired at a resolution of 15,000 (maximum ion injection times 60 ms). Repeated sequencing of peptides was minimized by setting a dynamic exclusion of 20 s.

Raw MS files were processed with MaxQuant (version. 1.6.1.3) [23]. The false discovery rate (FDR) cut-off was set to 1% for protein and peptide spectrum matches. Peptides were required to have a minimum length of 7 amino acids and a maximum mass of 4600 Da. Peak list files were searched against the UniprotKB Homo sapiens database, based on the 2018_02 release, combined with 245 common contaminants by the integrated Andromeda search engine [24]. The mass spectrometry proteomics data have been deposited to the ProteomeXchange Consortium via the partner repository with the dataset identifier PXD010513. Data analysis, statistics and annotation enrichment analysis was performed with the Perseus software package, version 1.5.4.2 [25]. Protein Groups were filtered for at least two valid values in at least one group of triplicates. Differentially expressed proteins were identified by t-test at a permutation-based FDR cut-off of 0.05, 250 randomizations and S0 = 0.5, which was used to determine the curves of the volcano plot. Pathway enrichment analysis was performed using Fisher exact test with a Benjamini-Hochberg FDR cutoff of 0.02. GOCC, GOBP, GOMF, CORUM, Uniprot Keywords and KEGG pathway annotations were used for the analysis. Bar plots were generated of significant protein of interest was performed by differential enrichment analysis of proteomic data, DEP [26].

### Transmission electron microscopy

Transmission electron microscopy (TEM) was performed on 31- and 56-day-old (post-plating) iPSC-CM from IsP and AuP DCM patients (n = 2 each for two clones) and from healthy controls (n = 2). The samples were fixed with 2.5% glutaraldehyde in 0.1 M cacodylate buffer, post fixed in potassium ferrocyanide reduced osmium, and further processed for epoxy resin (Agar100) embedded as previously reported [27]. The ultra-thin sections were cut with a diamond knife at 60 nm thicknesses using an EM UC7 Leica ultramicrotome (Leica Microsystems, Wetzlar, Germany) and double stained with 1% uranyl acetate and Reynolds lead citrate. TEM was performed using a Morgagni 286 transmission electron microscope (FEI Company, Eindhoven, The Netherlands) at 80 kV. Digital electron micrographs were recorded with a MegaView III CD and iTEM-SIS software (Olympus, Soft Imaging System GmbH, Münster, Germany). Digital electron micrographs were recorded with a MegaView III CD. From each sample, two separate EBs from each batch were examined under electron microscope. Ten CMs of each EBs were photographed. The measurements were performed to assess the length of sarcomeres and the size of the Z-bands (sarcomeres width). The measurements were done using iTEM-SIS software (Figs D and E in S1 File (Olympus, Soft Imaging System GmbH, Münster, Germany) and exported as Excel documents.

### Statistical analysis

Results are expressed as Mean±SEM and represent mean percentage of change (unless indicated otherwise). Data were analyzed with Sigmastat (Systat Software Inc., Chicago, Illinois) and Prism 5.0 (GraphPad Software, San Diego, California). P<0.05 was considered significant.

## Results

### The titin-mutated patients

The Israeli DCM patient (denoted IsP) was a 28-year old (at the time of skin biopsy) affected male described by Yoskovitz et al [9]. See On-line Supplement for clinical data. The mutation is an adenine insertion at position 86076 (c.86076dupA) causing a frameshift in the *TTN* gene. The Australian DCM patient (denoted AuP) was a 62 year old previously described by Gerull et al [28]. The mutation is a 2-bp insertion of adenine and thymidine at position 70690 (c.70690dupAT) leading to a frameshift with premature stop codon in the *TTN* gene. Clinical data are provided in the On-line Supplement.

### iPSC generation, characterization and differentiation into cardiomyocytes

The IsP iPSC clones 23.2 and 23.10 displayed normal karyotype (FigA in S1 File), expressed the pluripotent markers SSEA4, Oct4, TRA1-60 and Nanog (Panels A and B of FigB in S1 File) and demonstrated *in vivo* pluripotency (Panels C and D of FigB in S1 File). As seen in FigC in S1 File, the adenine insertion is present in the mutated fibroblasts and mutated iPSC clones, but absent in the healthy iPSC clone. The AuP iPSC and iPSC-CM were previously characterized by Gramlich and co-workers [10]. Table B in S1 File provides additional details on the patients (age, clinical characteristics), the generated iPSC (number of iPSC clones) and cardiomyocytes obtained and analyzed from each patient.

### Automaticity and the excitation-contraction coupling (ECC) machinery

#### Transmembrane action potential characteristics

**Fig 1:**
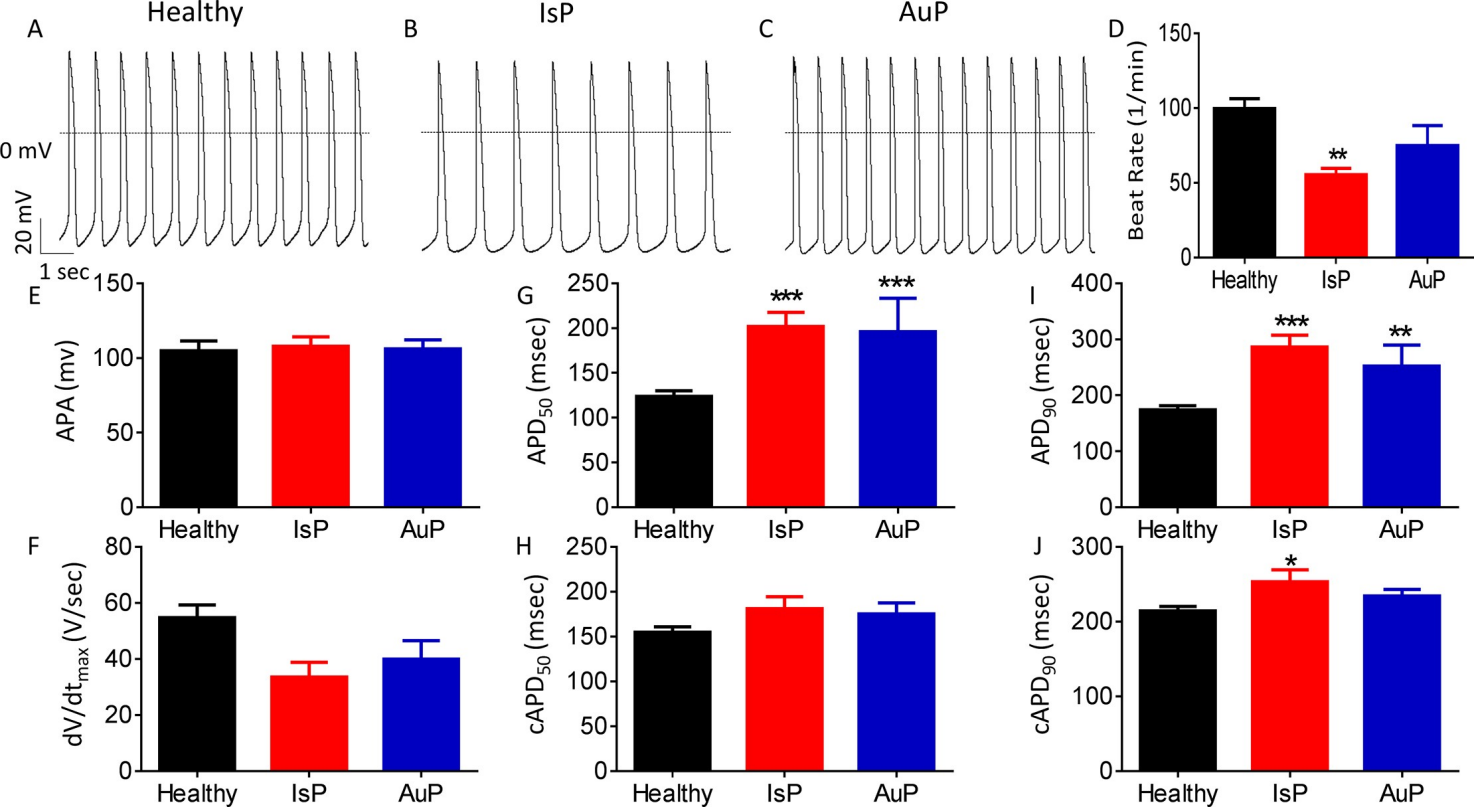
Action potential (AP) characteristics of healthy and mutated (IsP and AuP) iPSC-CM. [A-C] Recordings of spontaneous AP from healthy [A], IsP [B] and AuP [C] iPSC-CM. [D-J] Spontaneous AP parameters of healthy (38–77 day-old; black), IsP (54–77 day-old; red) and AuP (38–49 day old; blue) iPSC-CM: [D] beat rate, [E] action potential amplitude (APA), [F] maximum rate of phase 0 depolarization (dV/dt_max_), [G] and [I] action potential duration at 50% (APD_50_) and 90% (APD_90_) repolarization, respectively. [H] and [J] Corrected action potential duration at 50% (cAPD_50_) and 90% (cAPD_90_) repolarization, respectively. Healthy (clone KTI n = 23, clone KTN3 n = 30, n = 53); IsP (clone 23.2 n = 9, clone 23.10 n = 3, n = 12); AuP (n = 9). One-way ANOVA (on APA, dV/dt_max_, beat rate, APD_50_ and APD_90_) was performed followed by Holm-Sidak test, *P<0.05, **P<0.01 and ***P<0.001 vs healthy.

Firstly, we analyzed action potential (AP) characteristics in IsP, AuP and healthy spontaneously firing cardiomyocytes. While AP amplitude and maximal upstroke velocity of phase 0 depolarization (dV/dt_max_) were similar in IsP, AuP and healthy cardiomyocytes, the spontaneous firing rate was lower (P<0.01) in IsP iPSC-CM than control. In addition, APD at 50% and 90% repolarization (APD_50_ and APD_90_, respectively) were prolonged in IsP and AuP iPSC-CM compared to healthy iPSC-CM. However, when corrected to beat rate, the three groups showed no significant difference in APD_50,_ and in only APD_90_ of IsP iPSC-CM was slightly (by 39 ms) prolonged compared to control (Fig 1).

#### Automaticity, chronotropic β-adrenergic responsiveness and Beat Rate Variability (BRV)

**Fig 2:**
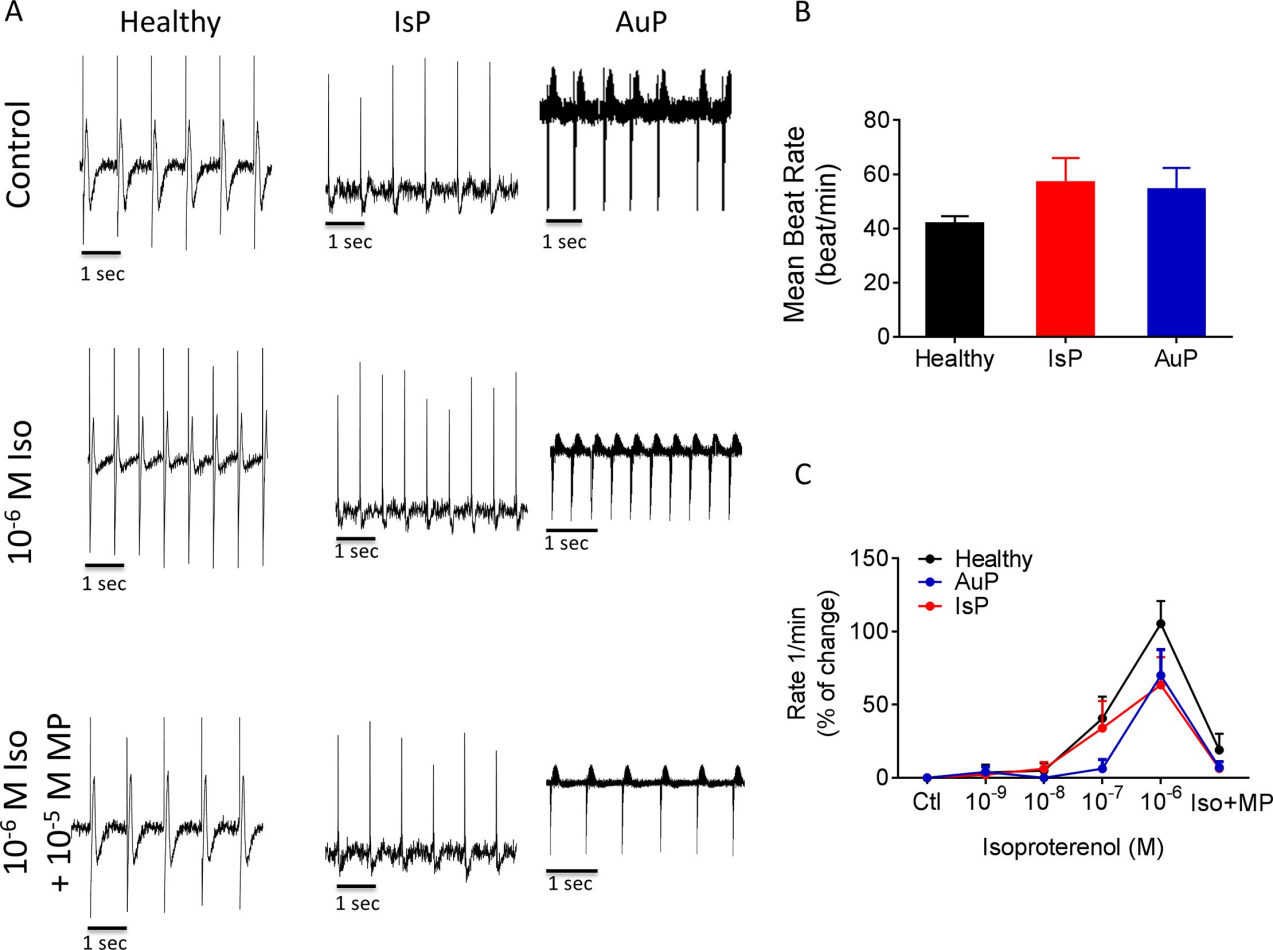
The chronotropic response to isoproterenol on spontaneous beat rate of healthy (black symbols), IsP (red symbols) and AuP (blue symbols) EBs. Panel [A] shows representative spontaneous electrogram recordings from healthy (left), IsP (middle) and AuP (right) EBs in the absence (upper) and presence (middle) of isoproterenol. The positive chronotropic effect of isoproterenol is blocked by the β-blocker metoprolol (lower). [B] The spontaneous beating rate of healthy (clone 24.5 n = 6, clone KTN3 n = 5, clone KTI n = 7, n = 18), IsP (clone 23.10 n = 5) and AuP (n = 3) EBs. [C] Summary of the response to isoproterenol of healthy (black), IsP (red) and AuP (blue) EBs. Results are expressed as Mean±SEM. There is no statistically significant difference between the groups in Two-way ANOVA test. Iso–isoproterenol, MP–metoprolol.

**Fig 3:**
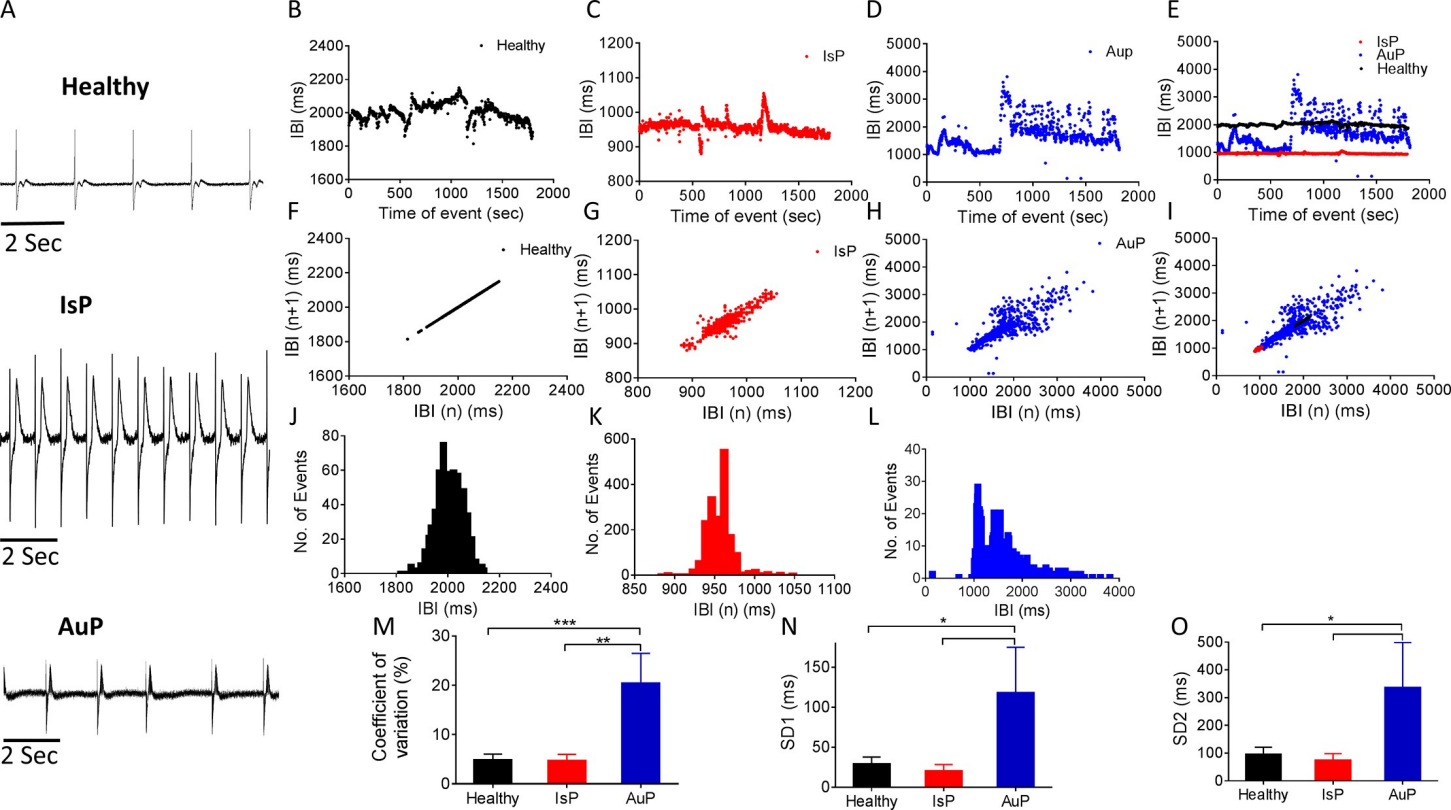
BRV characterization of EBs generated from healthy, IsP and AuP iPSC. [A] Representative electrogram recording from healthy, IsP and AuP iPSC-CM. [B-D] Representative IBIs time series of healthy (black), IsP (red) and AuP (blue) iPSC-CM and [E] combined IBIs time series. [F-H] Poincaré plots and [I] combined Poincaré plots of healthy (black), IsP (red) and AuP (blue) iPSC-CM. [J-L] histogram distribution of IBIs. [M-O] Summary of Coefficient of variation (COV) of IBIs (IBI CV) [M], SD1 [N] and SD2 [O] of Poincaré plots in healthy (clone 24.5 n = 6, clone KTN3 n = 5, clone KTI n = 6, n = 17), IsP (clone 23.10 n = 5) and AuP (n = 3) EBs. One-way ANOVA was performed followed by Holm-Sidak test, *P<0.05, **P<0.01 and ***P<0.001 *vs*. healthy.

In addition to the electrophysiological measurements at the cell level (Fig 1), we determined at the network level (a contracting EB) the spontaneous beat rate and the chronotropic response to β-adrenergic stimulation (Fig 2) as well as BRV characteristics, representing the non-linear firing patterns (Fig 3). As seen by representative electrograms (Fig 2A, upper row) and the summary (Fig 2B), the mean beat rate was similar in all 3 groups, suggesting that the titin mutation did not affect the basic mechanisms of automaticity. To determine whether the titin mutation altered the chronotropic responsiveness to β-adrenergic stimulation, we analyzed the effects of isoproterenol on the spontaneous beat rate of healthy (black), IsP (red) and AuP (blue) EBs. As illustrated in Fig 2A (middle row) and 2C, isoproterenol had a similar positive chronotropic effect in all 3 experimental groups, which was blocked by the β-blocker metoprolol (Fig 2A lower row).

As we previously showed that defective intracellular Ca^2+^ handling augments BRV magnitude [11,29], we compared BRV features between mutated and healthy EBs (Fig 3). The IBI time series (Fig 3B–3E) and IBIs histogram distribution (Fig 3J–3L) show that the IBI range of AuP EBs (~3000 ms) is broader than healthy (~400 ms) and IsP (~150 ms) EBs. The larger IBI dispersion of AuP EBs is also demonstrated by the much more scattered Poincaré plot (Fig 3F–3I). Accordingly, the BRV characteristics: Coefficient of variation (Fig 3M), and Poincaré plots SD1 (Fig 3N) and SD2 (Fig 3O) were larger in AuP than in healthy and IsP EBs.

#### [Ca^2+^]_i_ transient and contraction characteristics

**Fig 4:**
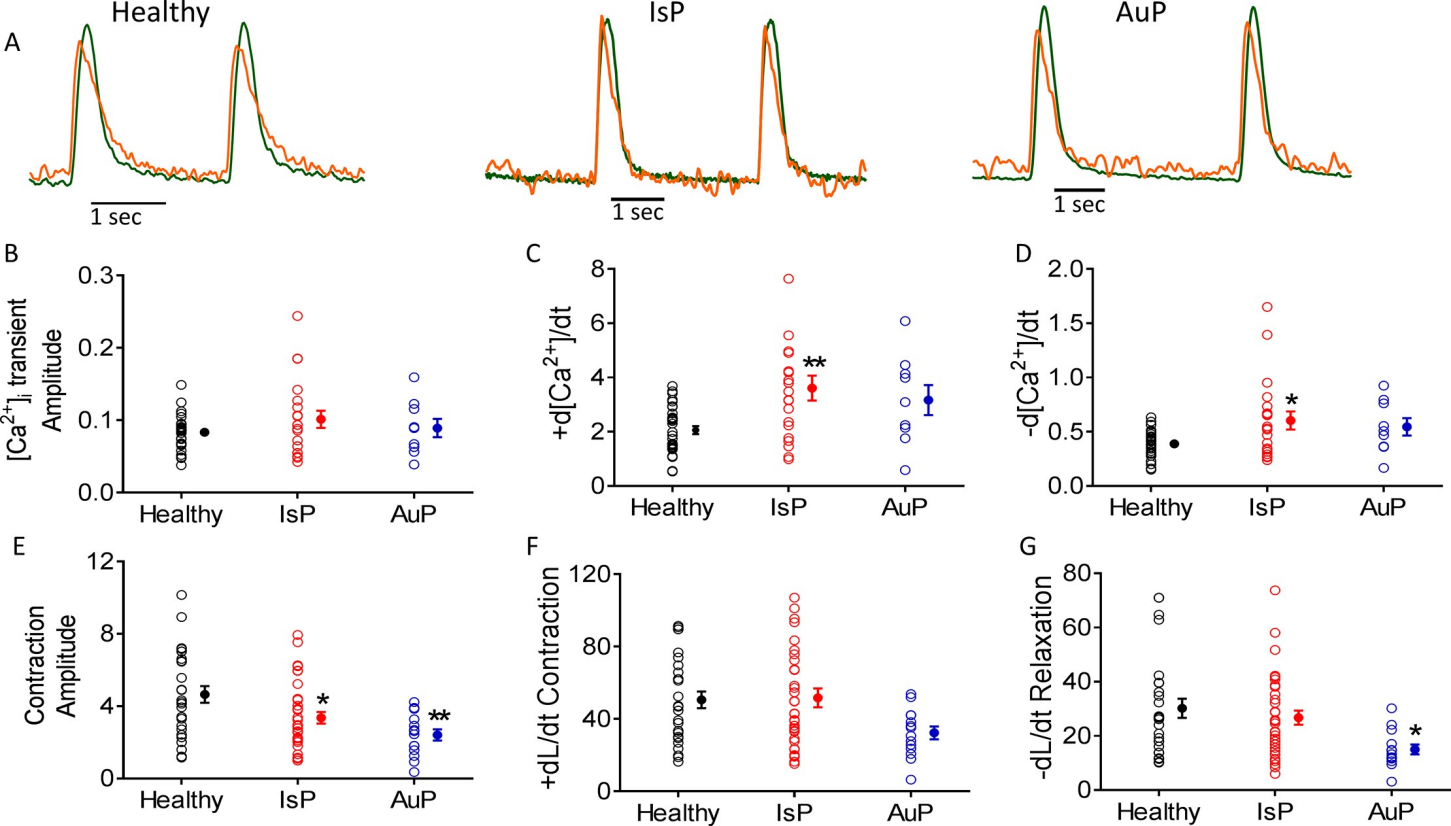
The [Ca^2+^]_i_ transient and contraction characteristics in healthy and mutated (IsP and AuP) iPSC-CM. [A] Simultaneous recording of [Ca^2+^]_i_ transient (orange) and contraction (green) measured from healthy (left), IsP (middle) and AuP (right) iPSC-CM. [B-D] [Ca^2+^]_i_ transient healthy (clone 24.5 n = 32), IsP (clone 23.2 n = 8, clone 23.10 n = 12 n = 20), (AuP n = 9) amplitude and maximal rates of [Ca^2+^]_i_ rise and decay, respectively. [E-G] Contraction healthy (clone 24.5 n = 27), IsP (clone 23.2 n = 16, clone 23.10 n = 19, n = 35), AuP (n = 14) amplitude and maximal rates of contraction and relaxation, respectively. Next to each column of individual values, the Mean+SEM (filled symbol) is shown. One-way ANOVA was performed followed by Holm-Sidak test, *P<0.05, **P<0.01 and ***P<0.001 *vs*. healthy.

Because titin is a key structural protein featuring several regulatory domains [7], we investigated whether the [Ca^2+^]_i_ transient and contraction characteristics are altered in the mutated cardiomyocytes representative recordings from the 3 groups are illustrated in Fig 4A. Except for the following changes, all other [Ca^2+^]_i_ transient and contraction characteristics were similar in the 3 groups: (1) The contraction amplitude of mutated iPSC-CM was lower (*P< 0.05, **P< 0.01; in IsP and AuP respectively) than in healthy iPSC-CM (Fig 4E). (2) AuP dL/dt_Relaxation_ was lower (*P< 0.05) than healthy and IsP iPSC-CM (Fig 4G). (3) IsP maximal rate of [Ca^2+^]_i_ rise (+d[Ca^2+^]_i_/dt) was higher (**P< 0.01) than in healthy iPSC-CM (Fig 4C). (4) IsP minimal rate of [Ca^2+^]_i_ rise (-d[Ca^2+^]_i_/dt) was higher (*P< 0.05) than in healthy iPSC-CM (Fig 4D).

### The responsiveness to positive inotropic interventions

As Gramlich et al demonstrated that exposure of mice with truncated titin to isoproterenol or angiotensin-II (AT-II) mimic typical features of DCM—left ventricular dilatation with impaired fractional shortening [30], we investigated whether the mutated and healthy cardiomyocytes respond differently to the following positive inotropic interventions: (1) β-adrenergic stimulation by isoproterenol; (2) elevated [Ca^2+^]_out_; (3) AT-II.

#### β-adrenergic stimulation

**Fig 5:**
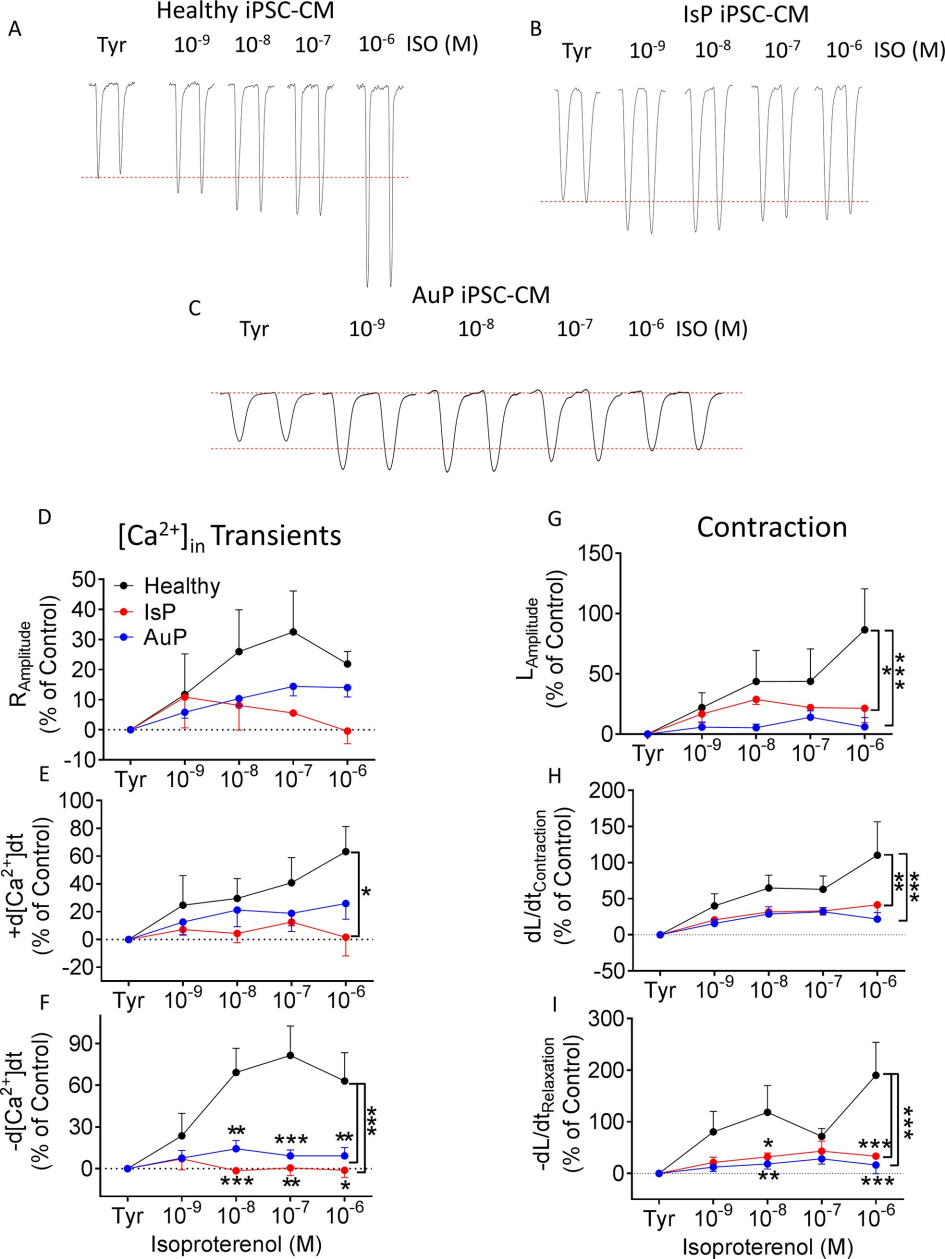
Effect of isoproterenol on contraction and [Ca^2+^]_i_ transient parameters of healthy and mutated iPSC-CM. [A-C] Representative contractions (L_Amplitude_) from healthy [A], IsP [B] and AuP [C] iPSC-CM in the absence and presence of isoproterenol. Panels [D-F] display the summary of [Ca^2+^]_i_ transient parameters of mutated (red–IsP, clone 23.2 n = 2, clone 23.10 n = 1, n = 3; blue–AuP, n = 8) and healthy (black–clone 24.5 n = 6) iPSC-CM: amplitude [D], and maximal rates of [Ca^2+^]_i_ rise [E] and decay [F]. Panels [G-I] show the reduced response of mutated iPSC-CM (red–IsP, clone 23.2 n = 6, clone 23.10 n = 2, n = 8; blue–AuP, n = 8) compared to healthy (black–clone 24.5 n = 6) iPSC-CM in all contraction parameters: amplitude [G], and maximal rates of contraction [H] and relaxation [I]. Results are expressed as percent change from control values in Tyrode’s solution. Two-way ANOVA was performed followed by Holm-Sidak test. Two-way ANOVA showed a statistically significant difference in all 3 contraction parameters between healthy and IsP groups (P<0.05). Statistically significant difference was also seen in maximal rate of [Ca^2+^]_i_ rise and relaxation between the 2 groups. For specific isoproterenol concentrations: *P<0.05, **P<0.01, ***P<0.001 vs Healthy. ISO–isoproterenol; Tyr–Tyrode’s solution.

A fundamental cardiac feature is β-adrenergic positive inotropic response caused by increased sarcoplasmic reticulum (SR) Ca^2+^ release through ryanodine receptor 2 (RyR2) channels [31]. This effect is demonstrated in healthy cardiomyocytes by a dose-dependent increase in [Ca^2+^]_i_ transient amplitude, maximal rate of [Ca^2+^]_i_ rise and decay of the [Ca^2+^]_i_ transient, contraction amplitude and maximal rates of contraction and relaxation (representative traces in Fig 5A, black symbols in Fig 5D–5I). In contrast, the mutated cardiomyocytes (representative traces in Fig 5B and Fig 5C for IsP and AuP, respectively) were barely responsive to isoproterenol respecting all measured parameters (red and blue symbols for IsP and AuP in Fig 5D–5I, respectively).

#### Elevated [Ca^2+^]_out_

**Fig 6:**
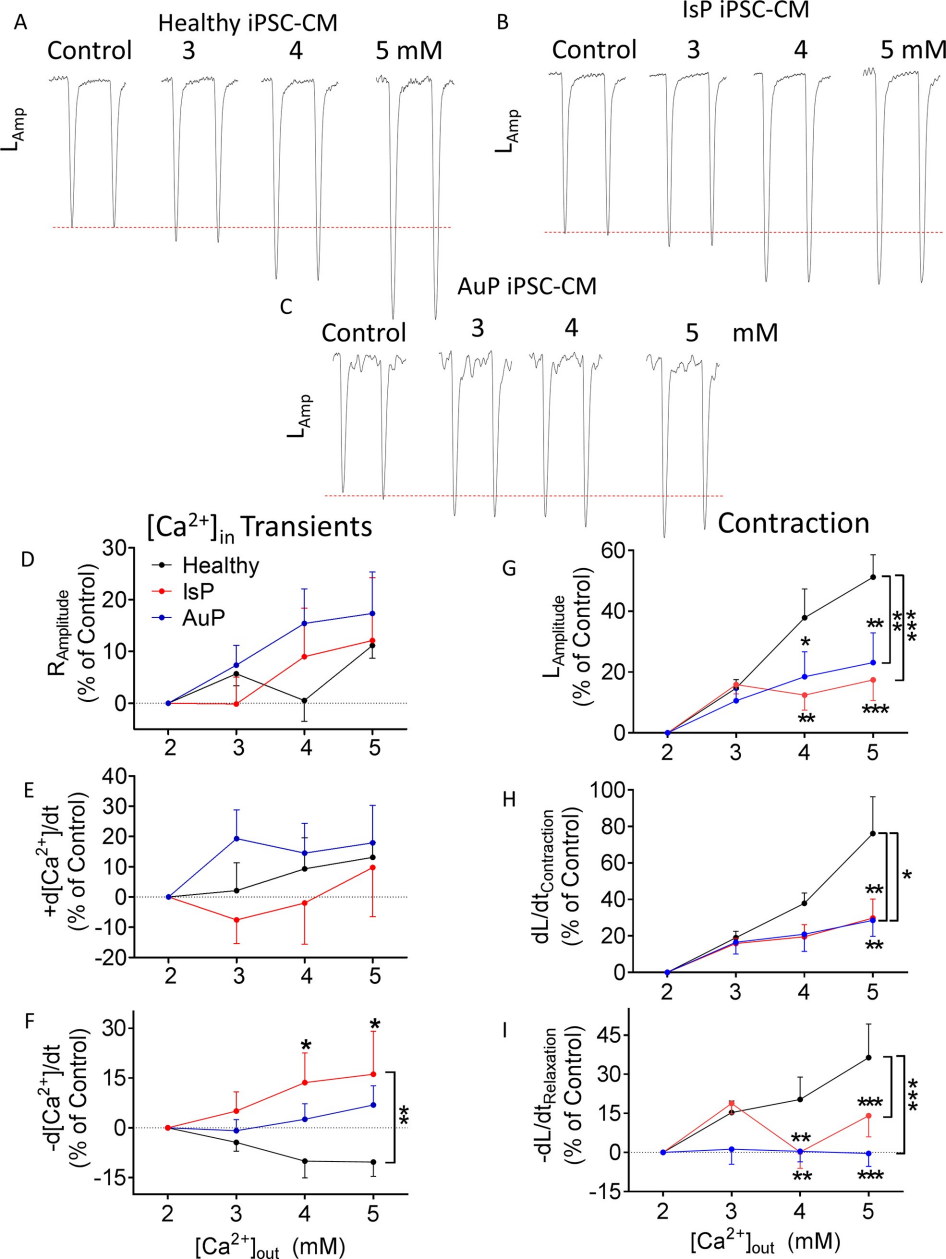
Effect of increasing [Ca^2+^]_out_ on contraction and [Ca^2+^]_i_ transient parameters of healthy and mutated iPSC-CM. [A-C] Representative contractions (L_Amplitude_) from healthy [A], IsP [B] and AuP [C] iPSC-CM in presence of increased [Ca^2+^]_out_. Panels [D-F] display the summary of [Ca^2+^]_i_ transient parameters of mutated (red–IsP, clone 23.2 n = 3, clone 23.10 n = 4, n = 7; blue–AuP, n = 8) and healthy (black–clone 24.5 n = 7) iPSC-CM: amplitude [D], and maximal rates of [Ca^2+^]_i_ rise [E] and decay [F]. Panels [G-I] show the reduced response of mutated iPSC-CM (red–IsP, clone 23.2 n = 7, clone 23.10 n = 11, n = 18; blue–AuP, n = 11) compared to healthy (black–clone 24.5 n = 12) iPSC-CM in all contraction parameters: amplitude [G], and maximal rates of contraction [H] and relaxation [I]. Results are expressed as percent change from control values at 2 mM. Two-way ANOVA was performed followed by Holm-Sidak test. Two-way ANOVA showed a statistically significant difference in all 3 contraction parameters between healthy and AuP groups and between healthy and IsP groups (P<0.05). Statistically significant difference was also seen in maximal rate of [Ca^2+^]_i_ relaxation between all 3 groups. For specific [Ca^2+^]_out_ concentrations: *P<0.05 vs Healthy, **P<0.01, ***P<0.001.

To decipher whether the attenuated response to isoproterenol was due to dysfunctional β-adrenergic cascade or alternatively–due to impaired downstream element mediating any positive inotropic intervention, we investigated the inotropic effect of elevating [Ca^2+^]_out_ (2, 3, 4 and 5 mM), which augments L type Ca^2+^ current (I_Ca,L_), in turn increasing SR Ca^2+^ release, thereby increasing contractile force [32]. As seen in Fig 6, while control cardiomyocytes were responsive to elevated [Ca^2+^]_out_, that of mutated cardiomyocytes was blunted. Specifically, all contraction parameters of IsP and AuP iPSC-CM were significantly lower than those of healthy iPSC-CM (Fig 6G–6I). In addition, there was no difference in [Ca^2+^]_i_ transient parameters between the 3 groups, except for maximal rate of [Ca^2+^]_i_ decay which was significantly higher (than control) in IsP cardiomyocytes (Fig 6D–6F).

#### Angiotensin-II (AT-II)

**Fig 7:**
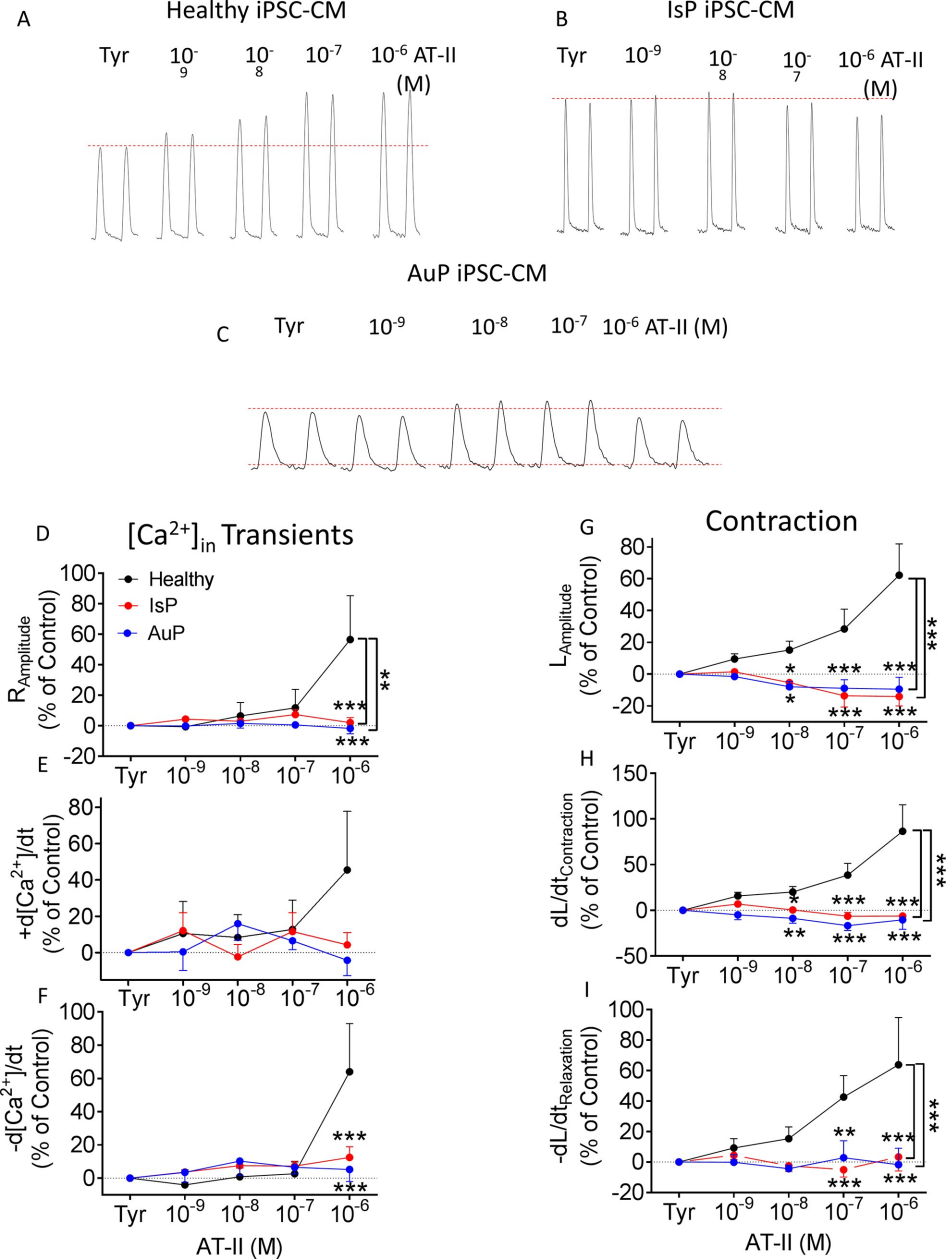
Effect of AT-II on contraction and [Ca^2+^]_i_ transient parameters of healthy and mutated iPSC-CM. [A-C] Representative contractions (L_Amplitude_) from healthy [A], IsP [B] and AuP [C] iPSC-CM in the absence and presence of AT-II. Panels [D-F] display the summary of [Ca^2+^]_i_ transients parameters of mutated (red–IsP, clone 23.2 n = 2, clone 23.10 n = 8, n = 10; blue–AuP, n = 5) and healthy (black–clone 24.5 n = 8) iPSC-CM: amplitude [D], and maximal rates of [Ca^2+^]_i_ rise [E] and decay [F]. Panels [G-I] show the reduced response of mutated iPSC-CM (red–IsP clone 23.2 n = 3, clone 23.10 n = 6, n = 9; AuP–blue, n = 5) compared to healthy (black–clone 24.5 n = 11) iPSC-CM in all contraction parameters: amplitude [G], and maximal rates of contraction [H] and relaxation [I]. Results are expressed as percent change from control values in Tyrode’s solution. Two-way ANOVA was performed followed by Holm-Sidak test. Two-way ANOVA showed a statistically significant difference in all 3 contraction parameters between healthy and IsP groups (P<0.001). Statistically significant difference was also seen in [Ca^2+^]_i_ transient amplitude between the 2 groups. For specific AT-II concentrations: **P<0.01, ***P<0.001 vs Healthy. AT-II–angiotensin-II; Tyr–Tyrode’s solution.

Based on the above mentioned findings we speculated that the SR–the downstream common step mediating these positive inotropic effectors, is defective in titin-mutated cardiomyocytes. To test this notion, we investigated whether AT-II, also inducing its positive inotropy via SR-Ca^2+^ release [21], is equally ineffective in mutated cardiomyocytes. Indeed, while in healthy iPSC-CM AT-II caused a prominent dose-dependent positive inotropic and lusitropic effects, markedly augmenting both [Ca^2+^]_i_ transient and contraction characteristics (Fig 7), mutated cardiomyocytes were unresponsive, collectively suggesting that a down-stream step mediating the positive inotropic and lusitropic effect is defected in the titin-mutated cardiomyocytes.

### Mechanisms underlying attenuated inotropic response of mutated iPSC-CM

To determine possible mechanisms underlying the depressed inotropic response of mutated iPSC-CM, we investigated: (1) structural features and sarcomeric organization; (2) The RyR-mediated SR Ca^2+^ release using caffeine; (3) The proteomic profile using MS-based shotgun proteomics.

#### Structural features and sarcomeric organization

To determine whether titin mutations are associated with ultrastructural changes, the Z-width measurements and sarcomere length analysis were performed on 30- and 60-day-old IsP (clones 23.2 and 23.10), AuP (clone T1) and healthy (clones 24.5 and KTN3) EBs. The sarcomere length analysis showed no significant differences between healthy and Aup and IsP cardiomyocytes, regardless of age (see Panels D and E of FigD and FigE in S1 File and raw data in Table A in S1 File). iPSC-CM showed a heterogeneous cell morphology, round-oval cells connected with elongated and stellate cells. Also iPSC-CM showed various amounts of primitive Z-dense bodies, nascent sarcomeres or primitive sarcomeres forming myofibrils of variable length. Healthy and mutated iPSC-CM contained myofibrils that showed poor alignment or organized sarcomeric pattern on all samples, consistent with the immature phenotype. The number of sarcomeres could not be appreciated with a degree of satisfactory accuracy because CM in EBs showed an irregular shape, with cellular extensions not trapped in the 60 nm section plane. Very few myofibrils developed A- and I-bands either at 30 days or 56 days post-plating from control and titin mutated EBs and this aspect could not be reliable quantified on ultrathin sections.

**Fig 8:**
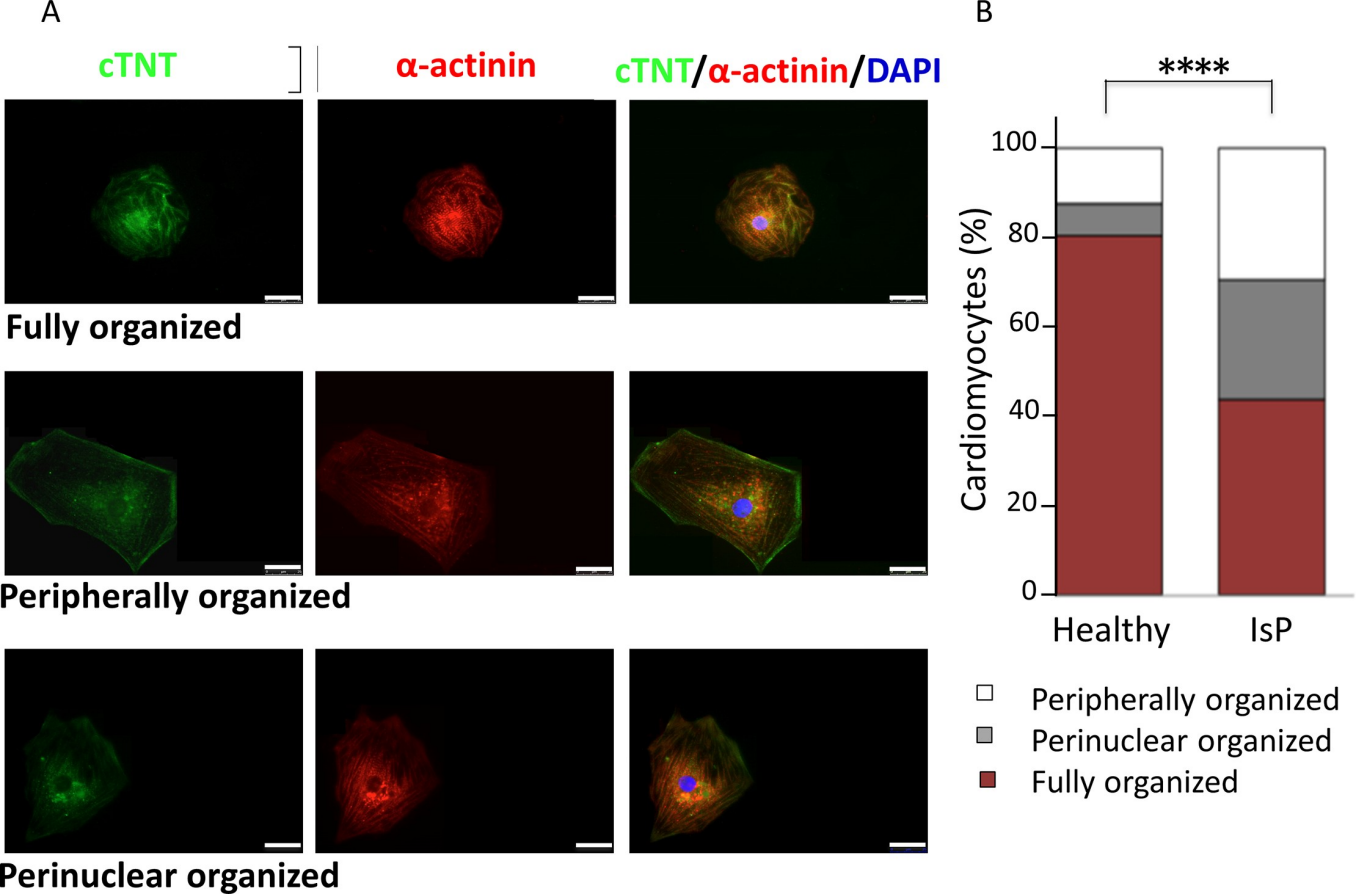
Levels of sarcomeric organization in control and IsP iPSC-CM. Panel [A] shows immunofluorescence images of α-actinin and cardiac troponin-T (cTNT) in healthy and IsP mutated single cardiomyocytes, illustrating three different levels of sarcomeric organization—fully, peripherally and perinuclear organized (upper, middle and lower panels, respectively). Panel [B] presents percentage of cells with different levels of sarcomeric organization. Statistic difference was tested using the chi-squared test Healthy (clone 24.5 n = 274), IsP (clone 23.2 n = 160, clone 23.10 n = 160, n = 320; ****P<0.001 Healthy vs IsP iPSC-CM). Scale bars = 25 μm.

Sarcomeric organization of mutated and healthy iPSC-CM was determined by co-staining of α-actinin (Z disk) and cardiac troponin-T (A-band) [10]. Briefly, single cardiomyocytes were dissociated from spontaneously beating embryoid bodies (EBs), and analyzed 7 days later. Cardiomyocytes were divided into 3 groups according to sarcomeric organization extending to: (1) the whole cytoplasm (Fig 8A, upper row); (2) <50% of the whole cytoplasm with main localization in the cell periphery (Fig 8A, middle row); (3) <50% of the whole cytoplasm with main localization in the perinuclear area (Fig 8A, lower row). As seen in Fig 8B, the percentage of cells with fully structured myofilaments occupying the entire cytoplasm is significantly reduced in IsP compared to healthy iPSC-CM (44% vs. 80%, respectively, P<0.0001). In contrast, in IsP iPSC-CM there is a higher percentage of cardiomyocytes in which organized myofibrils occupy only half of the cytoplasm or even less (P<0.0001). This group was further divided into two sub-groups according to the localization of the few organized myofibrils, which were visible either in the perinuclear area or at the cell periphery (Fig 8B). In the IsP group 26.6% of the cells show a sarcomere with an organized pattern visible only in the perinuclear area and 29.4% display an organized pattern limited at the cell periphery. The respective percentages in the healthy iPSC-CM were 7.3% and 12.4%, respectively. Importantly, these results are in agreement with the findings obtained by Gramlich et al in AuP cardiomyocytes (fully organized 54%, perinuclear 17%, and peripherally 29%) [10], suggesting the presence of titin mutation-related defects in both myofibril reassembly and sarcomere stability.

#### The RyR-mediated SR Ca^2+^ release using caffeine

**Fig 9:**
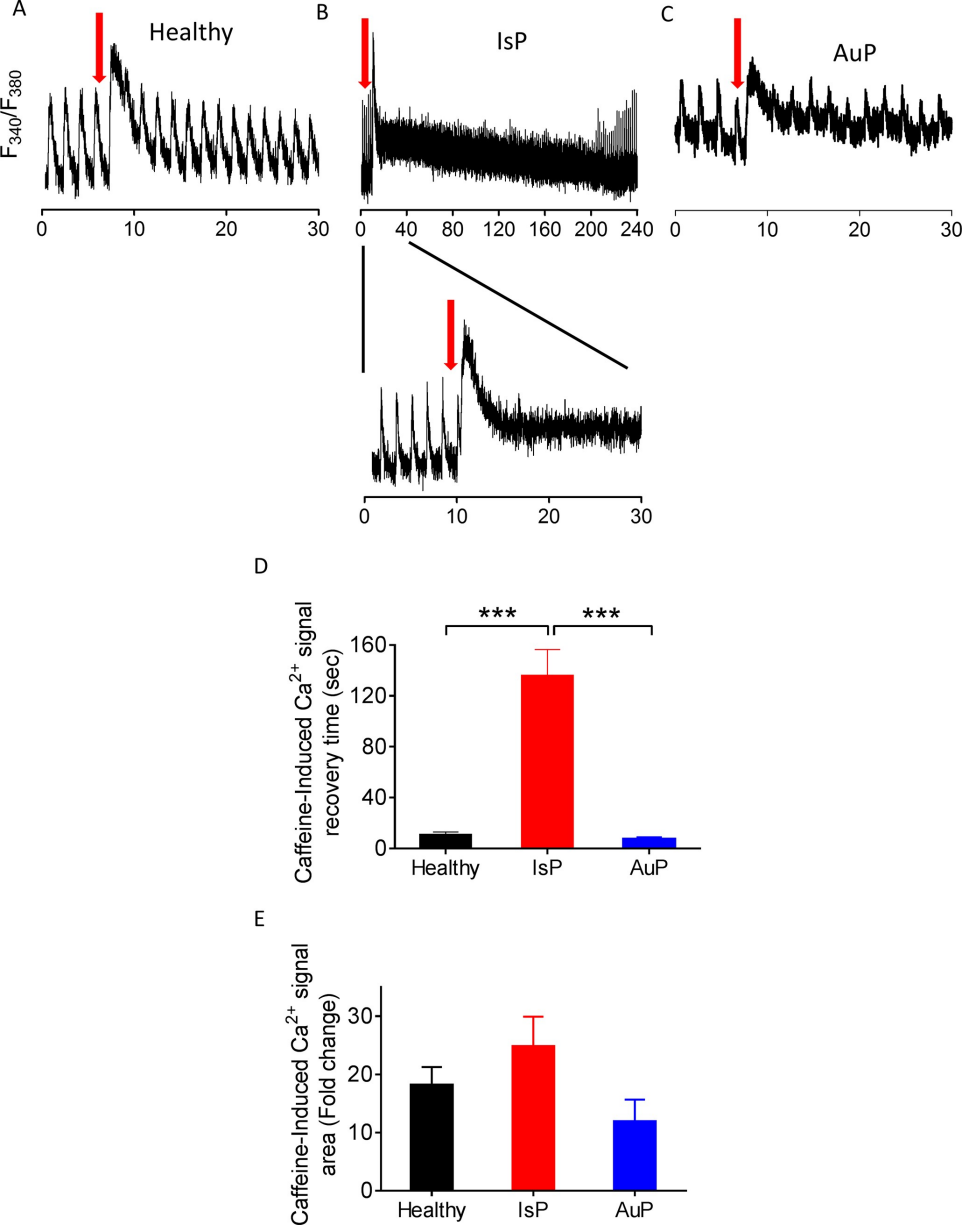
Effect of caffeine on [Ca^2+^]_i_ cycling of healthy and mutated iPSC-CM. Representative [Ca^2+^]_i_ transients from healthy [A], IsP [B] and AuP [C] iPSC-CM under caffeine administration (indicated by red arrow). The IsP cardiomyocytes display two-phase decline in [Ca^2+^]_i_ level: fast decline as in healthy cells, and gradual slow decline until reaching [Ca^2+^]_i_ basal level [B]. [D] Average recovery time from maximum of caffeine peak phase until the beginning of departing phase of the first measurable [Ca^2+^]_i_ transient post-caffeine insertion of healthy (24.5 clone n = 11), IsP (clone 23.2 n = 2, clone 23.10 n = 4, n = 6) and AuP (n = 8) iPSC-CM. [E] display the percent change in fold change in area of caffeine-induced [Ca^2+^]_i_ signal compared to the pre-caffeine [Ca^2+^]_i_ transient of healthy (clone 24.5 n = 11), IsP (clone 23.2 n = 2, clone 23.10 n = 4, n = 6) and AuP (n = 8) iPSC-CM. One-way ANOVA was performed followed by Holm-Sidak test, ***P < 0.001 (vs Control and AuP).

To decipher the mechanism(s) underlying the diminished response of titin-mutated iPSC-CM to all positive inotropic interventions operating by increasing [Ca^2+^]_i_, we tested the effect of a brief application of caffeine (10 mM, serves as an opener of the RyR2 receptor [12]) on the [Ca^2+^]_i_ transient. As illustrated by the representative experiments (Fig 9A–9C) and summary (Fig 9D and 9E), titin-mutated and healthy cardiomyocytes differed in their response to caffeine. As we previously reported [12], healthy iPSC-CM displayed an abrupt amplitude transient increase in intracellular Ca^2+^ concomitant with contraction cessation, followed by a decline in the Ca^2+^ level along resumption of contractions after ~10 sec (Fig 9A). In contrast, mutated iPSC-CM displayed prolonged recovery which was divided into two phases: (1) decline in [Ca^2+^]_i_ level as seen in healthy cardiomyocytes, but not to the basal level; (2) a gradual slow decline that lasts few minutes until reaching basal [Ca^2+^]_i_ level, during which resumption of contractions occurs (Fig 9B) (in IsP but not AuP cardiomyocytes). Consequently, IsP iPSC-CM recovery following caffeine administration was much slower compared to healthy iPSC-CM and the gradual decline in [Ca^2+^]_i_ level was longer (Fig 9D).

#### Protein expression analysis

**Fig 10:**
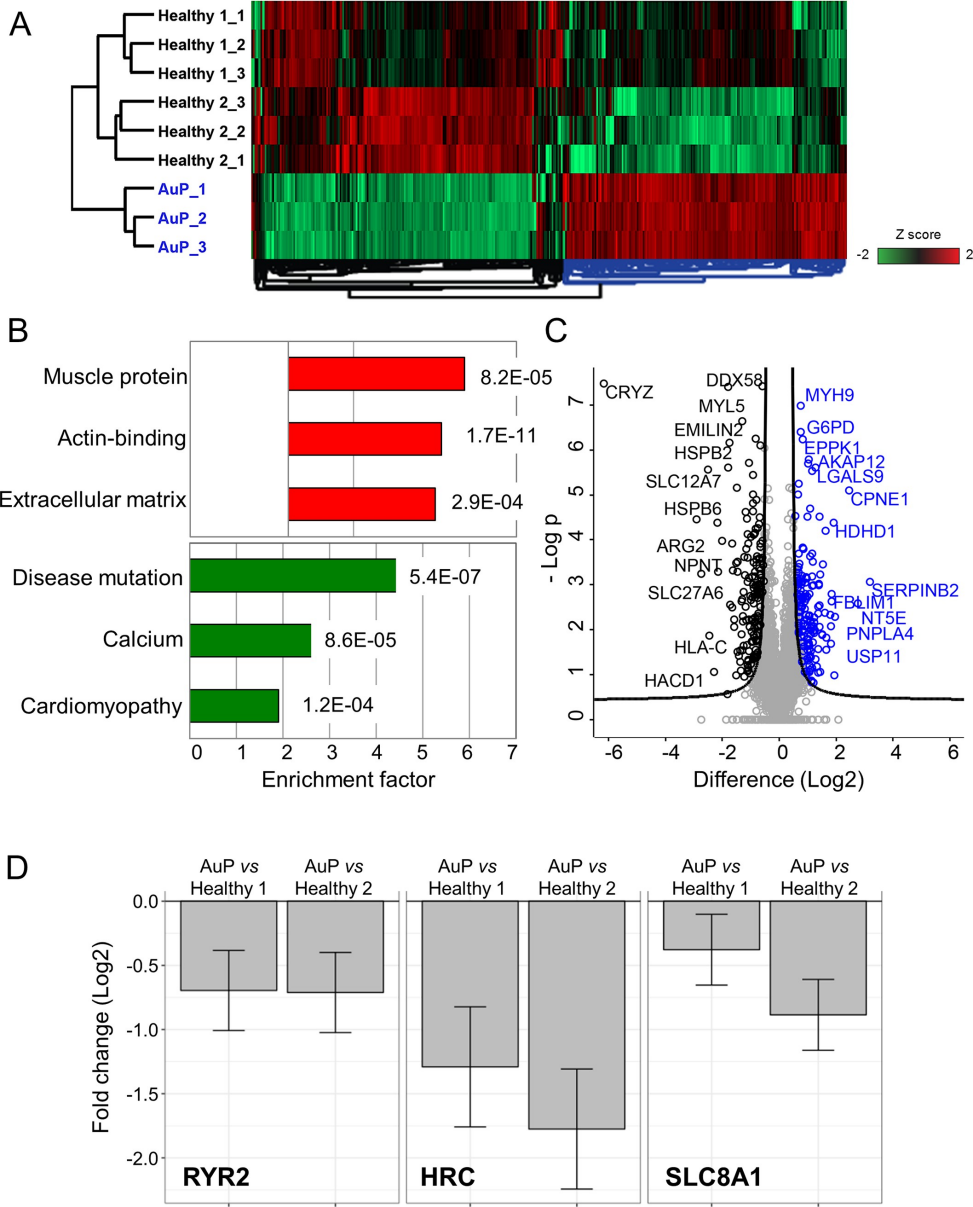
Mass spectrometry-based proteomic analysis of AuP and healthy cardiomyocytes. [A] Unsupervised hierarchical clustering of 301 proteins with significantly different expression in healthy (clone 24.5) and AuP cardiomyocytes (FDR 0.05, S0 0.5). Technical triplicates are shown for each sample. Healthy 1 and 2 indicate two unrelated control individuals. [B] Bar graphs showing the fold increase of the top three annotation enrichments in the cluster of proteins that are upregulated (red bars) and downregulated (green bars) in the AuP cardiomyocytes. The corresponding p value is reported next to each bar. [C] Volcano plot of statistical significance against fold-change (FDR 0.05, S0 0.5), highlighting the proteins with higher expression in control (black) and AuP (blue), respectively. [D] Bar plots depicting the log2 fold change expression of selected cardiac Ca^2+^ handling proteins that are differentially expressed between AuP and both healthy control cardiomyocytes.

Altogether, 301 unique proteins showed significant expression changes between AuP and healthy cardiomyocytes (151 upregulated and 150 downregulated; permutation-based FDR 0.05, S0 0.5) (Fig 10A). In the cluster of upregulated proteins, the top 3 significantly enriched keyword categories were Extracellular matrix, Actin-binding and Muscle protein, whereas Disease mutation, Calcium and Cardiomyopathy were identified as top significantly enriched Keyword terms within the downregulated proteins (Fig 10B). An additional detailed analysis of the proteome profile of the AuP cardiomyocytes revealed that indeed the expression levels of three important cardiac Ca^2+^ handling proteins (the ryanodine receptor 2—RYR2, the sarcoplasmic reticulum histidine-rich calcium-binding protein—HRC, and the sodium/calcium exchanger 1—SLC8A1) were significantly reduced in patient cells (Fig 10D).

## Discussion

To decipher the cellular mechanisms underlying the functional abnormalities, we investigated the actual mutated cardiomyocytes derived from DCM patients carrying titin mutations. The major findings were: (1) diminished response to isoproterenol, elevated [Ca^2+^]_out_ and AT-II; (2) altered response to caffeine, compared to healthy cardiomyocytes.

### Spontaneous electrophysiological features of titin-mutated iPSC-CM

Despite the statistically significant lower firing rate of IsP iPSC-CM and prolonged APD displayed by both IsP and AuP iPSC-CM, these differences lack practical significance since the mean firing rate and APD values fall well within the normal range previously described in iPSC-CM [29,33]. Additionally, when corrected to beat rate, the only significant difference in APD_90_ was between IsP iPSC-CM and control. Furthermore, this difference was less than 40 ms, leaving IsP iPSC-CM well within the normal range and thus lacking practical significance [33].

AuP iPSC-CM exhibited markedly increased BRV indices compared to healthy and IsP iPSC-CM. The mutation in both IsP and AuP patients deletes the A-band segment, leading to defective sarcomerogenesis through disruption of mechanical force transmission from myosin [34]. Currently, we do not know the reason for this difference in BRV parameters. Perhaps it is associated with disturbed mitochondrial Ca^2+^ handling machinery, which we previously showed to affect BRV magnitude [11].

### Attenuated responsiveness of mutated iPSC-CM to isoproterenol and [Ca^2+^]_out_

While the basal [Ca^2+^]_i_ transient and contraction parameters were mostly similar, the positive inotropic response of the mutated cardiomyocytes was markedly suppressed. While healthy cardiomyocytes exhibited the expected positive inotropic and lusitropic effects, the mutated cardiomyocytes displayed blunted response to isoproterenol (Fig 3). These findings are in agreement with 2 recent studies: (1) Hinson and co-workers showed that iPSC-CM generated from DCM patient carrying the pP22582fs+/- titin mutation (different than the AuP and IsP mutations) demonstrated diminished responses to β-adrenergic stress using contractile function assay [35]. (2) Peng et al reported diminished response to increased [Ca^2+^]_out_ and isoproterenol of cardiomyocytes derived from titin M-line deficient mice, which was attributed to reduction in expression levels of Ca^2+^ handling proteins SERCA2 and PLB [36]. In addition, reduction in protein expression levels was observed also for the Ca^2+^-binding protein calmodulin, which participates in SR Ca^2+^release and regulates the activity of SERCA2 and PLB through Ca^2+^/Calmodulin-dependent kinase II (CaMKII) [36]. Further details regarding the titin kinase domain are provided below.

Next, we subjected the healthy and mutated cardiomyocytes to increased [Ca^2+^]_out_. Positive inotropic and lusitropic effects in all 3 contraction parameters–amplitude, maximal rates of contraction and relaxation—were observed in healthy cardiomyocytes, as previously reported [32,36] (Fig 6). Nevertheless, IsP and AuP cardiomyocytes displayed reduced response to increased [Ca^2+^]_out_ compared to healthy cells. In contrast, only the maximal rate of [Ca^2+^]_i_ relaxation differed between the three groups.

### Impaired responsiveness of mutated iPSC-CM to AT-II and caffeine

Subsequent to the findings of decreased response of titin-mutated iPSC-CM to isoproterenol and [Ca^2+^]_out_, we sought to decipher the contribution of the SR to these findings. Therefore we investigated in healthy and mutated iPSC-CM the effect of AT-II, promoting synthesis of 1,4,5-inositol triphosphate (1,4,5-IP_3_) which triggers SR Ca^2+^ release [21]. Similarly to isoproterenol and [Ca^2+^]_out_, AT-II caused positive inotropic and lusitropic effects in healthy iPSC-CM on [Ca^2+^]_i_ transient and contraction parameters, as opposed to lack of response in IsP and AuP iPSC-CM (Fig 7). These results are consistent with results published by Gramlich et al [30]. The authors found that mice with truncated titin lacking part of the A-band and the M-band regions showed no alterations in cardiac morphology and function under normal conditions. However, when exposed to cardiac stress by means of isoproterenol or AT-II, these agents mimicked typical features of DCM—left ventricular dilatation with impaired fractional shortening.

To further investigate intracellular Ca^2+^ handling, we studied the effect of a brief application of caffeine (10 mM). Caffeine, being an opener of the RyR2 receptor, induces Ca^2+^ release from the SR which results in increased [Ca^2+^]_i_ levels [12]. IsP iPSC-CM showed longer recovery period until resumption of contraction compared to healthy and AuP iPSC-CM (Fig 9). Moreover, when compared to healthy cells, diastolic Ca^2+^ remained elevated in patient iPSC-CM after caffeine application, suggesting defects in SR calcium re-uptake and/or cellular Ca^2+^ efflux. Concordantly, proteomic analysis in AuP iPSC-CM revealed reduced level of the sarcoplasmic reticulum histidine-rich calcium-binding protein (HRC), a regulator of Ca^2+^ sequestration in the SR [37], and the sodium/calcium exchanger (SLC8A1), which represents the major Ca^2+^ efflux mechanism in cardiac myocytes (Fig 10D) [37]. Similar results were reported for cardiomyocytes derived from patients in a DCM family carrying a point mutation in cardiac troponin-T gene [38]. These cardiomyocytes displayed prolonged decay time in response to caffeine, suggesting altered function of Ca^2+^ pumps in the sarcolemma and SR membranes. Furthermore, DCM iPSC-CM displayed abnormal contractility which was rescued following overexpression of SERCA2, indicating compromised Ca^2+^ handling in DCM cardiomyocytes due to reduced expression of SERCA2 and other Ca^2+^ related elements [36,38]. In addition, we showed that in response to caffeine administration iPSC-CM generated from catecholaminergic polymorphic ventricular tachycardia type 2 (CPVT2) patients exhibited two-phase decline in Ca^2+^ level compared to healthy cells [12]. We suggested that the vast and prolonged Ca^2+^ release is due to the mutated calsequestrin protein in the CPVT2 patients which result in high levels of free Ca^2+^ in the SR, and not necessarily from a total higher SR Ca^2+^ levels [12]. Accordingly, altered function of Ca^2+^ handling protein such as calsequestrin and SERCA2 might be the reason for the abnormal response of IsP iPSC-CM in response to caffeine. This aspect is further detailed below.

### Structural analysis in titin-mutated cardiomyocytes

TEM analysis showed that both healthy and mutated iPSC-CM had nascent sarcomeres and disarrayed myofibrils with clear Z bands, with no ultrastructural differences between the three cell types in both age groups. Several studies reported on immature sarcomere and myofibrils structure in healthy cardiomyocytes seen in long-term culture up to 180 days [20,39]. Moreover, only in 360 day-old cardiomyocytes there were first signs of M-band [39]. An important study by Hinson et al demonstrated shorter sarcomere length in titin-truncated iPSC-CM compared to wild-type, but the authors used three-dimensional cardiomyocytes microtissues that can be possibly more mature than our cultured iPSC-CM [35]. Perhaps immaturity of the iPSC-CM is the reason for the presence of only clear Z bands in titin-mutated and healthy 31- and 56-day-old cardiomyocytes. Sarcomeric organization analysis showed defects in assembly and maintenance of a stable sarcomeric structure in mutated IsP compared with healthy iPSC-CM (Fig 8), which corresponds to the results of Gramlich et al in AuP iPSC-CM [10].

We performed sarcomeric organization analysis and found defects in assembly and maintenance of a stable sarcomeric structure in titin-mutated IsP iPSC-CM which lack the A-band segment. These results corresponds to those of Gramlich et al in AuP iPSC-CM which also lack the A-band segment [10]. In addition, Chopra et al found in titin-mutated iPSC-CM lacking A-band segment generated from DCM patients, that titin truncation mutations lead to defective sarcomere formation and myofibrillar assembly due to insufficient length of the titin protein [34]. Furthermore, Tonino et al showed in a mouse model in which part of the A-band was deleted that thick filament length was decreased in cardiac and skeletal muscles, and functional studies revealed reduced force generation and a DCM phenotype [40]. These findings suggest that titin truncation mutations leading to deletion of the A-band damage sarcomerogenesis through disruption of mechanical force transmission from myosin [34].

### Titin kinase domain

The titin protein is located within the sarcomere as a third filament connecting between Z-line and M-line. Between these edges, the titin is divided between the A-band and the elastic and extensible I-band. We performed sarcomeric organization analysis and found defects in assembly and maintenance of a stable sarcomeric structure in titin-mutated IsP iPSC-CM which lack the A-band segment. These results correspond to those of Gramlich et al in AuP iPSC-CM which also lack the A-band segment. In addition, Chopra et al found in titin-mutated iPSC-CM lacking A-band segment generated from DCM patients, that titin truncation mutations lead to defective sarcomere formation and myofibrillar assembly due to insufficient length of the titin protein. Furthermore, Tonino et al showed in a mouse model in which part of the A-band was deleted that thick filament length was decreased in cardiac and skeletal muscles, and functional studies revealed reduced force generation and a DCM phenotype. These findings suggest that titin truncation mutations leading to deletion of the A-band damage sarcomerogenesis through disruption of mechanical force transmission from myosin.

In addition to its structural role, titin participates in various signal-transduction pathways through several sites, specifically the titin kinase (TK) domain located at the M-band [41,42]. Several studies investigated TK and its diverse signaling pathways and interactions, and found that it is involved in muscle mechanical signaling [42–45]. An important study by Peng et al investigated the effect of TK absence on cardiac function using M-line deficient mice lacking TK [36]. On the cellular level, there was no difference in sarcomere structure between 30-day old control and M-line deficient cardiomyocytes, similar to our results obtained from ultrastructure analysis. Only after 80 days in culture, M-line deficient cardiomyocytes displayed sarcomere disassembly. Additionally, cardiac function was investigated, and under basal conditions there was no difference between 30-day old control and M-line deficient cardiomyocytes, similar to our findings of contraction parameters. Moreover, the β-adrenergic stimulator dobutamine which causes a positive inotropic effect, caused blunted response in M-line deficient cardiomyocytes compared to control, in agreement with our findings in titin-mutated cardiomyocytes [36]. The authors suggested that the mechanism underlying the reduced contractility of M-line deficient cardiomyocytes, is associated with calcium handling machinery, and is based on protein expression levels analysis in which early reduction of SERCA2 and PLB levels was observed. These results may indicate that titin serves as a contractility regulator through its TK effects on calcium handling proteins [36].

The above mentioned findings indicate TK importance in myofibrillar mechanical signaling, contractile function and cardiac gene expression. These findings may constitute the basis for the results obtained from IsP and AuP titin-mutated cardiomyocytes, which lack the M-line region and specifically the TK domain. Furthermore, our results are consistent with results previously published by Gramlich and co-workers [30]. This group found that mice with truncated titin lacking the TK show no alterations in cardiac morphology and function under normal conditions. However, when exposed to cardiac stress by means of isoproterenol or AT-II, they mimic typical features of DCM—left ventricular dilatation with impaired fractional shortening. More importantly, Gramlich et al (2015) generated iPSC-CM from Australian DCM patient (AuP) which lack the TK domain due to titin truncation mutation in exon 327 [10]. Significant down regulation of α- and β- myosin heavy chain and cardiac α-actin was observed in mutated cells compared with control, similar to previously published results [10,45].

These findings strengthen the notion that titin plays a key role in various cellular pathways, in addition to its role as molecular spring. Specifically, the TK domain is crucial for sarcomere assembly and stability, mechanosensory processes and regulation of gene expression through interactions with diverse proteins. Truncation of TK domain in mutant iPSC-CM may be a plausible mechanistic link to explain the observed results; however, this hypothesis was not addressed in the present study.

Altogether, we investigated iPSC-CMs from an Israeli (IsP) and Australian (AuP) titin-mutated (carrying different mutations) patients. All the findings on the IsP iPSC-CM are new. A recent study [10] by Moretti, Gramlich (co-authors on this manuscript) and co-workers using AuP iPSC-CM demonstrated that: (1) Correction of *TTN* reading frame in patient-specific cardiomyocytes derived from induced pluripotent stem cells rescued defective myofibril assembly and stability and normalized the sarcomeric protein expression (2) AON treatment in *TTN* knock-in mice improved sarcomere formation and contractile performance in homozygous embryos and prevented the development of the DCM phenotype in heterozygous animals. (3) Disruption of the *TTN* reading frame due to a truncating DCM mutation can be restored by exon skipping in both patient cardiomyocytes *in vitro* and mouse heart *in vivo*, indicating RNA-based strategies as a potential treatment option for DCM. All other findings regarding action potential characteristics, beat rate variability, ultrastructural analysis, inotropic responsiveness to AT-II and [Ca ^2+^]_out_, response to caffeine and proteomics, are completely novel.

In summary, our findings reflect an underlying abnormal contraction and calcium handling mechanism, which can be attributed to lack of titin kinase domain in the DCM patients. The kinase domain is involved in regulation of cardiac gene expression through interactions with MURF proteins, as was reported by several studies [36,43–45], and this involvement might contribute to the reduced response of titin-mutated iPSC-CM to positive inotropic interventions. Additional research is required to decipher the specific mechanism(s) responsible for the reduced contractility of IsP and AuP titin-mutated cardiomyocytes.

## Supporting information

S1 File Supplement. (DOCX)
