# Correction: Functional abnormalities in induced Pluripotent Stem Cell-derived cardiomyocytes generated from titin-mutated patients with dilated cardiomyopathy

**DOI:** 10.1371/journal.pone.0207548

**Published:** 2018-11-08

**Authors:** The PLOS ONE Staff

In the Author Contributions section, Michaela Gherghiceanu (MG) should have the contribution “Formal analysis” instead of “Resources”.

The following information is missing from the Funding section: This study was supported by the Rappaport Family Institute for Research in the Medical Sciences [grant number 015.03–01]".

The publisher apologizes for the errors.
