# Supplementary material for: Functional abnormalities in induced Pluripotent Stem Cell-derived cardiomyocytes generated from titin-mutated patients with dilated cardiomyopathy
Source: PLoS One. 2018 Oct 17;13(10):e0205719. doi: 10.1371/journal.pone.0205719 (PMC6192629; doi:10.1371/journal.pone.0205719)
Supplement: S1 File — (DOCX) [file pone.0205719.s001.docx]

**Functional abnormalities in induced Pluripotent Stem Cell-derived cardiomyocytes generated from titin-mutated patients with dilated cardiomyopathy**

Revital Schick^1,2,3¶^, Lucy N. Mekies^1,2,3¶^, Yuval Shemer^1,2,3^, Binyamin Eisen^1,2,3^, Tova Hallas^1,2,3^, Ronen Ben Jehuda^1,2,3,4^, Meital Ben-Ari^1,2,3^, Agnes Szantai^1,2,3,5^, Lubna Willi^1,2,3^, Rita Shulman^1,2,3^, Michael Gramlich^6^, Luna Simona Pane^7^, Ilaria My^8^, Dov Freimark^9^, Marta Murgia^10,11^, Gianluca Santamaria^12^, Mihaela Gherghiceanu^13^, Michael Arad^9^, Alessandra Moretti^8,14^, Ofer Binah^1,2,3^

^1^Department of Physiology, Biophysics and Systems Biology, Technion, Haifa, Israel.

^2^The Rappaport Institute, Haifa, Israel.

^3^Rappaport Faculty of Medicine, Technion, Haifa, Israel.

^4^Department of Biotechnology, Technion, Haifa, Israel.

^5^Department of Biochemistry, University of Szeged, Szeged, Hungary.

^6^Department of Cardiology and Cardiovascular Diseases, Eberhard Karls University, Tübingen, Germany.

^7^Discovery Biology, Discovery Sciences, IMED Biotech Unit, AstraZeneca, Gothenburg, Sweden.

^8^Medical Department – Cardiology, Klinikum rechts der Isar – Technische Universität München, Munich, Germany.

^9^Heart Failure Institute and Leviev Heart Center, Sheba Medical Center and Sackler School of Medicine, Tel Aviv University, Tel Aviv, Israel.

^10^Department of Proteomics and Signal Transduction, Max-Planck-Institute of Biochemistry, Martinsried, Germany.

^11^Department of Biomedical Sciences, University of Padova, Via Ugo Bassi, 58/B, 35131, Padua, Italy.

^12^Department of Experimental and Clinical Medicine, University of Magna Grecia, Medical School, Viale Europa Germaneto, 88100 Catanzaro, Italy.

^13^Victor Babes National Institute of Pathology, Bucharest, Romania.

^14^German Centre for Cardiovascular Research – partner site Munich Heart Alliance, Munich, Germany.

* Corresponding author

Email: [binah@tx.technion.ac.il](mailto:binah@tx.technion.ac.il) (OB)

^¶^These authors contributed equally to the manuscript.

**Supplementary Results**

**The Israeli patient (IsP)**

The patient was asymptomatic at age 28 during family screen and had normal ECG, left ventricular (LV) diastolic dimension (LVEDD) of 60 mm and left ventricular ejection fraction (LVEF) of 47%. Two years later he suffered from effort dyspnea and had LVEF of 35-40%. At 2012 at age 35 he was assessed as New York Heart Association class 3 (NYHA III) with considerable dyspnea in any activity and orthopnea. The LVEDD was 53 mm but his LVEF decreased to 33%. ECG examination revealed sinus rhythm, narrow QRS and low voltage in the limb leads. Holter recordings showed sinus rhythm with multiple premature ventricular contractions without complex arrhythmia, ventricular tachycardia or conduction block. A primary prevention ICD was implanted. His therapy is comprised of carvedilol, ramipril, spironolactone, furosamide and digoxin. In 2014, echocardiography (echo) evaluation showed LVEDD of 56 mm, LVEF of 30% and mild-moderate tricuspid regurgitation. Stress echo (terminated due to fatigue after 3 min of Bruce protocol) was characterized by a flat pulse and blood pressure response to exercise and no improvement in left ventricular contraction during stress.

**The Australian patient**

The patient (denoted AuP) is a 62 year old (at the time of skin biopsy) affected female member of an Australian family with familial DCM previously described by Gerull et al [1]. The mutation is a 2-bp insertion of adenine and thymidine at position 43,628 (c.43628insAT) leading to a frameshift with premature stop codon in the *TTN* gene. The patient was presented with autosomal dominant DCM with CHF, but without cardiac conduction defects or clinically detectable skeletal muscle disease. The patient began treatment with perindopril in 1996 due to mildly reduced LV systolic function. In June 2006 she had a syncopal episode with diaphoresis. Echo evaluation revealed low-normal LV systolic function (LVEF 50-55%) and a 24-hour Holter monitor examination was normal with 84 (<1%) VE’s and 7 (<1%) SVE’s. Electrophysiological study in 2007 revealed no inducible arrhythmias and normal conduction intervals. Her echo demonstrated LVEF of 40% and consequently carvedilol was added to perindopril. Subsequent echo revealed low-normal LVEF (50-55%) which remained at this level since then. In December 2014 her ECG revealed sinus rhythm with normal PR, QRS and QT intervals. She had 1^st^-degree LBBB, inferior Q waves and mild T wave inversion in V1 to V3. Holter monitor examination revealed sinus rhythm with rare VE's and SVE's (45 in total over 22 hours). Her most recent echo in December 2014 revealed normal LV size (LVDD 49 mm) with low-normal LVEF (50%) on medical therapy (carvedilol 25 mg bd, perindopril 5 mg daily). Both Australian and Israeli mutations are located in exon 154 of the N2B isoform or MetaExon 326 which is crucial to TTN structure being expressed in all isoforms except the small NOVEX-3 (PSI 100%).

**Titin-mutated iPSC generation, characterization and differentiation into functional cardiomyocytes**

The Israeli patient’s fibroblasts were reprogrammed using the STEMCCA cassette and iPSC clones 23.2 and 23.10 were generated. Karyotype of both clones was analyzed and found normal (SA Fig). The same procedure was performed for skin (clone 24.5) and hair (clones KTN and KTI) (previously described [2,3]) biopsies obtained from healthy subjects as control. Next, the iPSC clones were characterized for their pluripotency and were confirmed to express the typical pluripotent markers Nanog, Tra1-60, Oct4 and SSEA4 (SB Fig Panels A and B). Furthermore, the iPSC clones demonstrated *in vivo* spontaneous differentiation into derivatives of all three germ layers, using the teratoma assay, presented in SB Fig (Panels C and D). The Australian patient fibroblasts were reprogrammed using the Sendai virus as described in Gramlich et al [4]. Table B provides additional details regarding the patients and their generated iPSC clones: age, clone names, number of differentiations and clinical status.

**Genotyping the titin mutation**

The IsP titin mutation which causes the DCM was found by Yoskovitz et al to be an adenine insertion at position 58,880 (c.58880insA), causing a frame shift and resulting in a stop codon and protein truncation after 19,628 amino acids [5]. In order to confirm that the mutation is preserved in the iPSC clones, sequence analysis was performed on PCR products obtained from fibroblasts and the two iPSC clones generated from IsP DCM patient. As seen in SC Fig, the adenine insertion is present in the mutated fibroblasts obtained from the DCM patient (SC Fig Panel B) and in both mutated iPSC clones (SC Fig Panels C and D), but is absent from the healthy iPSC clone (SC Fig Panel A).

**Z-width measurements and sarcomere length analysis of healthy and titin-mutated cardiomyocytes**

The Z-width measurements and sarcomere length analysis were performed on 30- and 60-day-old IsP (clones 23.2 and 23.10), AuP (clone T1) and healthy (clones 24.5 and KTN) EBs. The sarcomere length analysis showed no significant differences between healthy and Aup and IsP cardiomyocytes, regardless of age (see SD Fig Panels D and E). However, the Z-width measurements showed significant differences between the AuP and healthy clone (24.5 clone) (**p< 0.01) in 30-day- old (see SD Fig Panel B), and between the IsP (23.10 clone) and healthy clone (*p< 0.05) in 60-day-old (see SD Fig Panel C). In addition, Table A displays the mean sarcomere lengths of the EBs, illustrating no statistical difference between mutated and healthy EBs in both age groups. In summary, the TEM analysis showed no ultrastructural differences between healthy and mutated samples from both DCM patients, regardless of age (see representative pictures for IsP, AuP and healthy cardiomyocytes (clones 24.5 and KTN) in SE Fig).

**Supplementary tables and figures:**

**SA Table: Mean sarcomere lengths of IsP (clones 23.2, 23.10), AuP (clone T1) and healthy (clones 24.5, KTN) EBs aged 31 and 56 days.**

| **Clone** | **Day** | **Sarcomere length (μm)** |
| --- | --- | --- |
| 23.2 (IsP) | 31 | 1.53±0.18 |
|  | 56 | 1.47±0.14 |
| 23.10 (IsP) | 31 | 1.53±0.19 |
|  | 56 | 1.46±0.18 |
| T1 (AuP) | 31 | 1.52±0.12 |
|  | 56 | 1.53±0.09 |
| KTN3 (Control) | 31 | 1.57±0.09 |
| 24.5 (Control) | 56 | 1.45±0.17 |

**SB Table: Characteristics of the patients.**

| **Patient** | **Age** | **Clones** | | **Number of differentiations** | | **Clinical status** |
| --- | --- | --- | --- | --- | --- | --- |
| IsP | 28 | 23.2 | 23.10 | >3 | >3 | DCM, LVEF=30%, ICD |
| AuP | 62 | T1 | | >3 | | DCM, LVEF=50% |

**SA Fig**


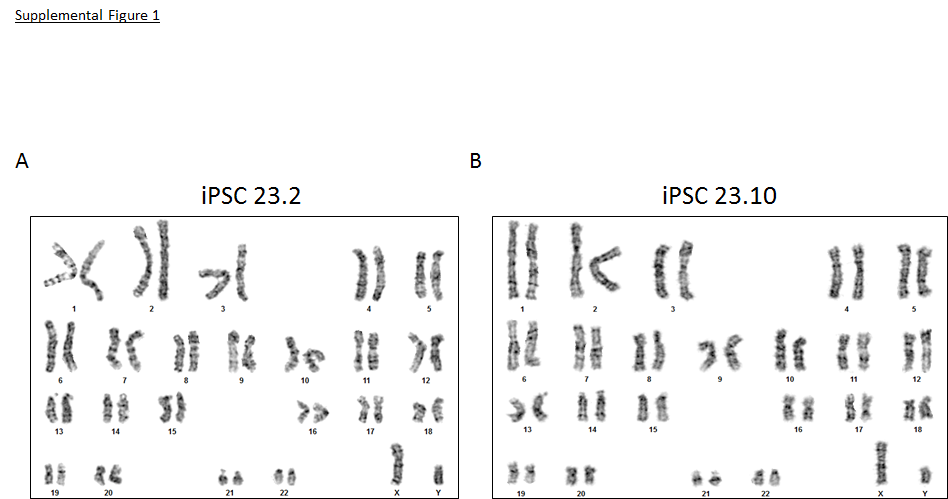


**Fig A.** **Karyotype analysis of the IsP iPSC clones 23.2 (panel A) and 23.10 (panel B), derived from IsP DCM patient.**

**SB Fig**

**
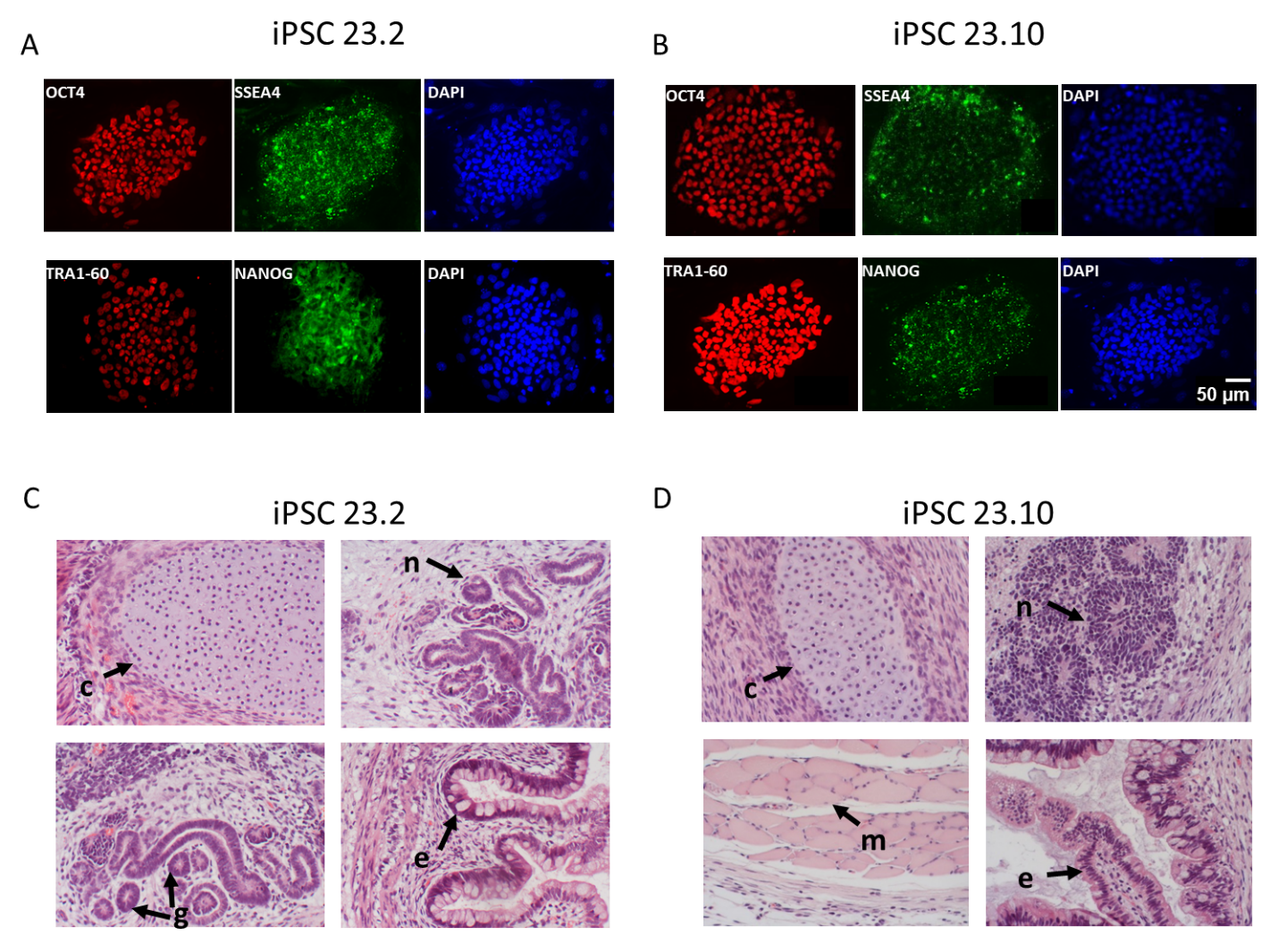
**

**Fig B. Pluripotency characterization of iPSC derived from the IsP DCM patient - Immunofluorescence staining [A, B] and histological analysis of teratomas [C, D].** Immunofluorescence staining of typical pluripotent markers shown for iPSC generated from IsP patient (panel A clone 23.2, panel B clone 23.10). Teratomas were obtained from SCID mice injected with IsP iPSC clones 23.2 (panel C) and 23.10 (panel D), which differentiated *in vivo* into derivatives of all three germ layers (ectoderm, mesoderm and endoderm). Ectoderm; n – neural tissue. Mesoderm; m – muscle, c – cartilage. Endoderm; e – epithelial cells, g – endocrine gland**.**

**SC Fig**

**Fig C. Genotyping the titin mutation Titin^insA^.** Sequence chromatograms from a healthy non-carrier iPSC (WT/WT) [A], IsP DCM patient’s fibroblast (M/WT) [B], and from two IsP iPSC clones (M/WT) 23.2 [C] and 23.10 [D]. The red circles in [B-D] mark the heterozygous adenine insertion in the mutated cells between nucleotides A and T (green circle in [A]), which causes a shift to the right of single nucleotide in the mutated strand, hence the nucleotide mix between the WT and mutated strands seen in the mutated chromatograms.

**SD Fig**


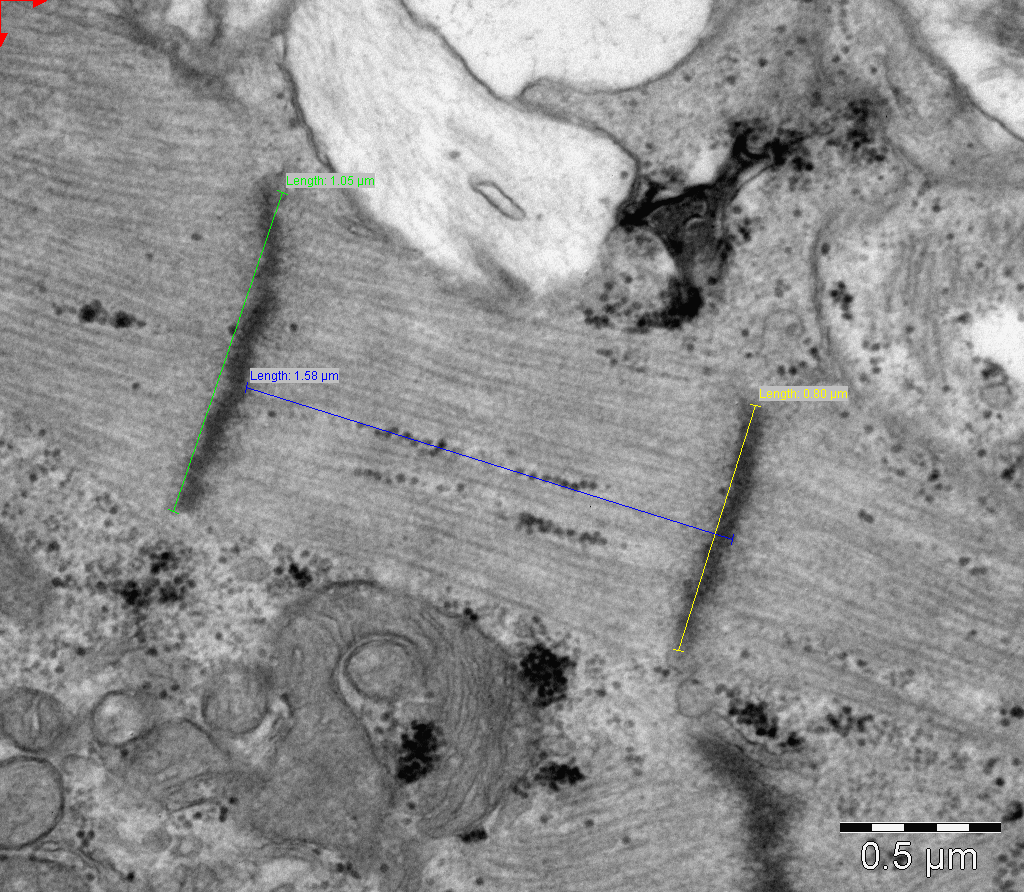


**
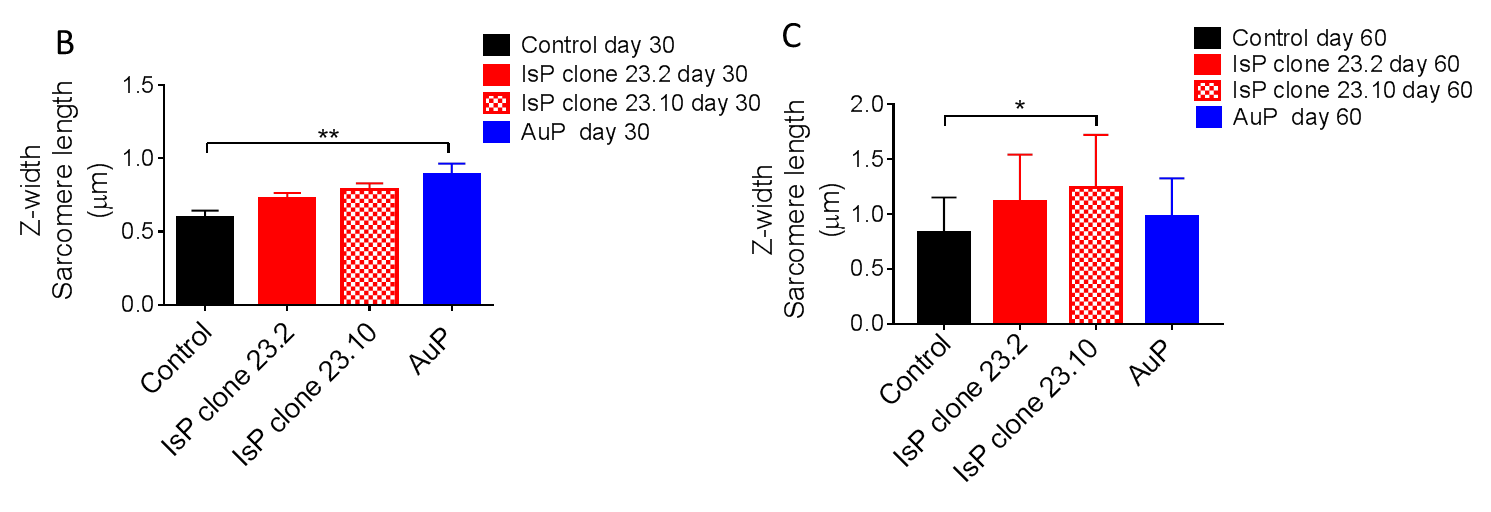
**

**
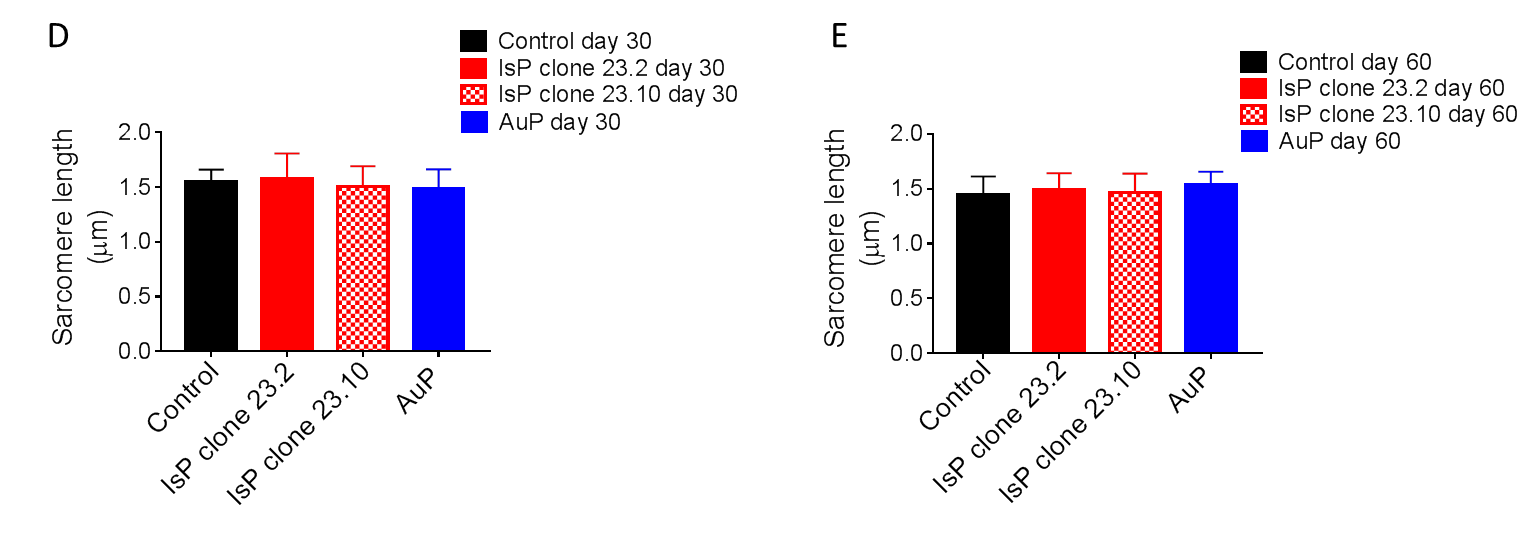
**

**Fig D. Transmission electron microscopy (TEM) showing Z-width measurements and sarcomere length.** [A] TEM image showing the method for measuring the length of a sarcomere and width of Z-bands, using Olympus iTEM software. [B] Z-width measurements of IsP (clones 23.2 and 23.10), AuP and healthy iPSC-CM (24.5 clone) in 30 days. This analysis shows significant difference between AuP and healthy iPSC-CM. Healthy n=20; IsP clone 23.2 n=20; IsP clone 23.10 n=20; AuP n=20. [C] Z-width measurements of IsP (clones 23.2 and 23.10), AuP and healthy iPSC-CM in 60 days. This analysis shows significant difference between IsP (23.10 clone) and healthy iPSC-CM. Healthy n=20; IsP clone 23.2 n=20; IsP clone 23.10 n=20; AuP n=20. [D-E] Sarcomere length of titin-mutated and healthy iPSC-CM is not significantly different at both 30 and 60-day-old cells respectively. Healthy n=40; IsP clone 23.2 n=40; IsP clone 23.10 n=40; AuP n=40. One-way ANOVA was performed followed by Holm-Sidak test, *p<0.05 and **p<0.01 *vs.* healthy.

**SE Fig**

**Fig E. TEM analysis of healthy and mutated iPSC-CM from two DCM patients.** The TEM analysis shows no ultrastructural differences between samples of healthy clones 24.5 and KTN [A, B] and titin mutated iPSC-CM from IsP clones 23.2 [C, D] and 23.10 [E, F], and AuP clone [G, H]. Clear defined sarcomeres with Z bands (Z) are visible at 31 days [A, C, E, G] and 56 days [B, D, F, H].

**References**

1. Gerull B, Gramlich M, Atherton J, McNabb M, Trombitás K, Sasse-Klaassen S, et al. Mutations of TTN, encoding the giant muscle filament titin, cause familial dilated cardiomyopathy. Nat Genet. 2002;30: 201–204. doi:10.1038/ng815

2. Ben-Ari M, Schick R, Barad L, Novak A, Ben-Ari E, Lorber A, et al. From beat rate variability in induced pluripotent stem cell-derived pacemaker cells to heart rate variability in human subjects. Heart Rhythm. 2014;11: 1808–18. doi:10.1016/j.hrthm.2014.05.037

3. Novak A, Shtrichman R, Germanguz I, Segev H, Zeevi-Levin N, Fishman B, et al. Enhanced Reprogramming and Cardiac Differentiation of Human Keratinocytes Derived from Plucked Hair Follicles, Using a Single Excisable Lentivirus. Cell Reprogramming (Formerly “Cloning Stem Cells”). 2010;12: 665–678. doi:10.1089/cell.2010.0027

4. Gramlich M, Pane LS, Zhou Q, Chen Z, Murgia M, Schötterl S, et al. Antisense-mediated exon skipping: a therapeutic strategy for titin-based dilated cardiomyopathy. EMBO Mol Med. 2015;7: 562–76. doi:10.15252/emmm.201505047

5. Yoskovitz G, Peled Y, Gramlich M, Lahat H, Resnik-Wolf H, Feinberg MS, et al. A novel titin mutation in adult-onset familial dilated cardiomyopathy. Am J Cardiol. Elsevier Inc.; 2012;109: 1644–1650. doi:10.1016/j.amjcard.2012.01.392
